# Next-Generation *Bacillus anthracis* Live Attenuated Spore Vaccine Based on the *htrA*^-^ (High Temperature Requirement A) Sterne Strain

**DOI:** 10.1038/srep18908

**Published:** 2016-01-06

**Authors:** Theodor Chitlaru, Ma’ayan Israeli, Erez Bar-Haim, Uri Elia, Shahar Rotem, Sharon Ehrlich, Ofer Cohen, Avigdor Shafferman

**Affiliations:** 1 Department of Biochemistry and Molecular Genetics, Israel Institute for Biological Research, Ness-Ziona 74100, Israel

## Abstract

Anthrax is a lethal disease caused by the gram-positive spore-producing bacterium *Bacillus anthracis*. Live attenuated vaccines, such as the nonencapsulated Sterne strain, do not meet the safety standards mandated for human use in the Western world and are approved for veterinary purposes only. Here we demonstrate that disrupting the *htrA* gene, encoding the chaperone/protease HtrA (High Temperature Requirement A), in the virulent *Bacillus anthracis* Vollum strain results in significant virulence attenuation in guinea pigs, rabbits and mice, underlying the universality of the attenuated phenotype associated with *htrA* knockout. Accordingly, *htrA* disruption was implemented for the development of a Sterne-derived safe live vaccine compatible with human use. The novel *B. anthracis* SterneΔ*htrA* strain secretes functional anthrax toxins but is 10–10^4^-fold less virulent than the Sterne vaccine strain depending on animal model (mice, guinea pigs, or rabbits). In spite of this attenuation, double or even single immunization with SterneΔ*htr*A spores elicits immune responses which target toxaemia and bacteremia resulting in protection from subcutaneous or respiratory lethal challenge with a virulent strain in guinea pigs and rabbits. The efficacy of the immune-protective response in guinea pigs was maintained for at least 50 weeks after a *single* immunization.

Anthrax is a severe disease caused by the gram-positive spore-producing bacterium *Bacillus anthracis*. Spores, which represent the infective form of the bacteria, are characterized by a strong resistance to environmental insults^1^. In nature, the spores mainly infect grazing herbivores by ingestion or inhalation. Human cases of anthrax due to exposure to infected animals or animal products were frequent in the past but are currently extremely rare in the Western world. However, the development of countermeasures for the prevention and treatment of anthrax has increased in recent years because of the potential malicious use of *B. anthracis* spores as a bio-terror weapon. This concern placed *B. anthracis* at the top of the CDC list of select agents (http://www.bt.cdc.gov/agent/agentlist-category.asp).

## Anthrax virulence factors

The most lethal manifestation of *Bacillus anthracis* infection is represented by the respiratory form of the disease: the inhaled spores are transported by alveolar macrophages and dendritic cells to lymph nodes where they germinate into fast growing vegetative bacilli^2^. The lethality of anthrax results from the bacterial exotoxins as well as the remarkable ability of the bacteria to proliferate in the host. *B. anthracis* secretes Lethal Toxin (LT) and Edema Toxin (ET), which are composed of binary combinations of the three proteins Protective Antigen (PA), Lethal Factor (LF) and Edema Factor (EF). These three proteins are encoded by genes located on pXO1, one of the two virulence plasmids naturally harboured by *B. anthracis*. PA, the common subunit of both toxins, is harmless by itself but plays the essential role of intracellular translocation of the lethal subunits of the toxin complex-LF (a zinc protease, which together with PA forms the exotoxin LT) and EF (an adenylate cyclase, which together with PA constitutes the exotoxin ET). An additional virulence factor is the poly D-glutamate anti-phagocytic capsule synthesized by enzymes encoded by genes located on the second native plasmid pXO2^3^,^4^.

## The HtrA (High Temperature Requirement A) protein of ***B. anthracis***

We have previously conducted functional-genomic and proteomic global surveys, combined with serological evaluation of *B. anthracis* antigens, for the identification of novel virulence factors as well as vaccine and diagnostic marker candidates among extracellular (secreted or membrane) proteins. These studies revealed a list of novel antigens (other than the classic exotoxins LT and ET) for further evaluation based on their immunogenicity, abundance under various culture conditions, and functional relatedness to infection^5^,^6^,^7^,^8^,^9^,^10^,^11^,^12^. HtrA, one of the *B. anthracis* proteins that emerged from these screens, is a secreted and membrane-associated bifunctional chaperone/protease encoded by a chromosomal gene. HtrA was subsequently shown to be abundantly expressed during infection and was consequently able to serve as an early disease-biomarker of anthrax^13^. Our recent study of the phenotype associated with directed disruption of the *htrA* gene^14^,^15^ demonstrated that HtrA is necessary for tolerance to a variety of stress stimuli and for the secretion of several major extracellular proteins. Most notably, *htrA* gene disruption in the fully virulent toxinogenic and capsular Vollum strain resulted in a dramatic attenuation in the guinea pig model following subcutaneous or intranasal exposure in spite of the fact that the mutated bacteria were able to express the PA, LF and EF toxin subunits as well as the anti-phagocytic capsule^15^. The extent of the virulence attenuation associated with *htrA* disruption was significantly higher than that promoted by disruption of any other reported *B. anthracis* virulence determinants (other than the LT and ET toxins and the capsule biosynthetic operon themselves), which were suggested to play a role in bacterial pathogenesis (examples of such factors are listed in Table 1). As shown in Table 1, the impact on virulence associated with a particular mutation was not manifested in all the animal models employed and by all the routes of exposure. Here, it is shown that this is not the case with the *htrA* disruption: infection studies of the *htrA*-disrupted *B. anthracis* fully virulent Vollum strain in three rodent models of anthrax demonstrate the universality of the attenuated phenotype.

Immunization of guinea pigs with sub-lethal doses of the *htrA*-disrupted *B. anthracis* Vollum strain elicited an immune response that conferred protection to a subsequent lethal challenge^15^. These results suggested that HtrA is a major virulence factor essential for manifestation of *B. anthracis* pathogenesis and that *htrA*-gene disruption may represent the basis for the development of novel prophylactics against *B. anthracis* infection.

## Anthrax vaccines

Because PA, the non-detrimental toxin subunit, is a very potent immunogen and its neutralization by the humoral immune response prevents the onset of the disease, all anthrax vaccine preparations contain PA^3^,^16^. The anthrax protein vaccines that are licensed for human use include partially purified PA preparations potentiated by various adjuvants.

In general, live attenuated vaccines are considered to promote superior protective immunity compared to sub-unit vaccines owing to the notion that exposure of the host to the infective micro-organism in the context of infection (i) elicits a response against a wide range of antigens rather than a unique immunogen, and/or (ii) involves a variety of immune and inflammatory mechanisms rather than a limited humoral response to one particular antigen. Attenuated toxin-producing live bacteria (such as the nonencapsulated Sterne strain, pXO1^+^; pXO2^−^) have been used worldwide as effective veterinary vaccines since the 1930 s and in the past were used for human vaccination. While millions of people were vaccinated with the Sterne-like live vaccine (known as the STI strain)^17^ between 1950 and 1980 in the former Soviet Union, the potential reactogenicity of the Sterne vaccine as indicated by animal experimentation, precluded use in humans in the Western world. Consequently, on grounds of toxicity, these live attenuated vaccines do not meet the safety standards mandated for human use and are suitable for veterinary purposes only. Yet, the protection conferred by administration of the Sterne attenuated live vaccine to experimental animals appeared to be superior to that provided by vaccination with PA in terms of the longevity of the protective response, efficacy against a broad spectrum of *B. anthracis* strains and the need for less administrations (1–2 injections, compared to the initial cumbersome 6-dose vaccination schedule with the subunit PA vaccine)^18^. Therefore, further attenuation of a Sterne vaccine strain could be a beneficial step in the development of next-generation live anthrax vaccines for human use.

The present study demonstrates, in three animal models, that disruption of the *htrA* gene in the *B. anthracis* Sterne-vaccine platform is sufficient to generate a highly attenuated strain that preserves its ability to induce a protective immune response.

## Results

### Virulence attenuation of the Vollum Δ*htrA* strain in various animal models of anthrax

**Figure 1:**
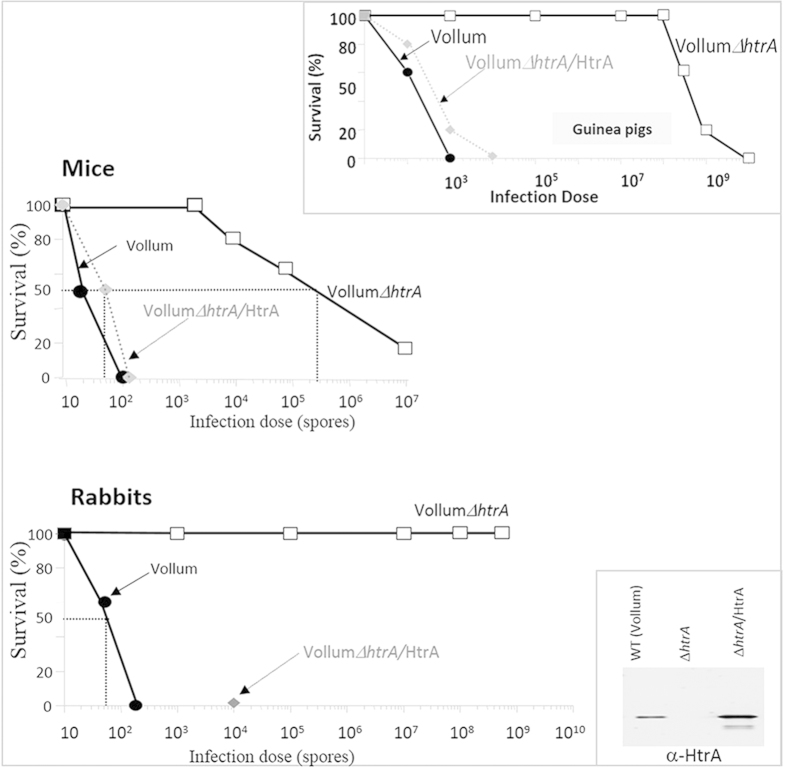
Survival profiles of mice and rabbits infected subcutaneously (SC) with the Vollum, VollumΔ*htrA* and transcomplemented VollumΔ*htrA*/HtrA strains. The animal groups (10 mice or 5 rabbits per experimental group) were inoculated SC with increasing spore doses of the parental Vollum (black circles), mutated VollumΔ*htrA* (white squares) and transcomplemented VollumΔ*htrA*/HtrA (grey diamonds/grey dotted line) strains. Animal survival was followed for 3 weeks. In the experiment carried out in rabbits, only the 10^4^ spore dose of the transcomplemented VollumΔ*htrA*/HtrA strain was tested, and all the animals in this experimental group succumbed to the infection. Upper-right inset: The results of a published previously similar experiment carried out with guinea-pigs^15^. A horizontal 50% survival dotted line in the “Mice” and “Rabbits” survival-profile panels indicates the estimated LD_50_ values of the various strains. Lower-right inset: Anti-HtrA Western blot analysis of the secretomes of the three strains demonstrating complete elimination of HtrA expression in the mutated strain and restoration of the HtrA level in the trans-complemented strain is shown.

To determine whether disruption of the *htrA* gene is associated with virulence attenuation not only in guinea pigs^15^ (see also Fig. 1, inset) but also in other animal models, studies assessing the survival of experimental animals following administration of the VollumΔ*htrA* strain were carried out in mice and rabbits. The experiments consisted of subcutaneous (SC) administration of increasing doses of either one of 3 strains of bacteria: the parental wild-type Vollum strain (pXO1^+^, pXO2^+^), its mutated Δ*htrA* derivative strain and the HtrA trans-complemented strain VollumΔ*htrA*/HtrA. The results depicted in Fig. 1 and summarized in Table 2 clearly demonstrate that in all three animal models the disruption of the *htrA* gene is associated with a substantial decrease of virulence. The attenuation indices (ratio between the LD_50_ values of the mutant and the wild-type parental strains) promoted by the *htrA* disruption in the Vollum strain background ranged from 10^4^ in mice to greater than 10^7^ in rabbits. These attenuation values are comparable to those observed in guinea pigs (Table 2). The extent of the virulence attenuation in mice is a rather unexpected result considering the fact that ICR out-bred mice exhibit high sensitivity to *B. anthracis* (as opposed to some other in-bred murine strains^19^) and that mice in general are notoriously sensitive to the poly-D glutamic acid capsule, the synthesis of which is not affected in the Vollum mutant strain^15^. This observation strongly supports the notion that HtrA is essential for the manifestation of *B. anthracis* virulence and underlines the dominant nature of the *htrA* mutation as a means for virulence attenuation. In the rabbit system, which is considered to represent a suitable animal anthrax model that more faithfully emulates the human disease^3^,^20^, none of the animals succumbed upon administration of as much as 5 × 10^8^ spores (higher doses were not attempted, therefore the LD_50_ value is considered to be above this dose). The virulence attenuation phenotype of VollumΔ*htrA* was restored to levels comparable to those of the parental Vollum strain upon expression of HtrA in the trans-complemented strains VollumΔ*htrA*/HtrA (Fig. 1).

### Generation and phenotypic characterization of the SterneΔ*htrA* strain

**Figure 2:**
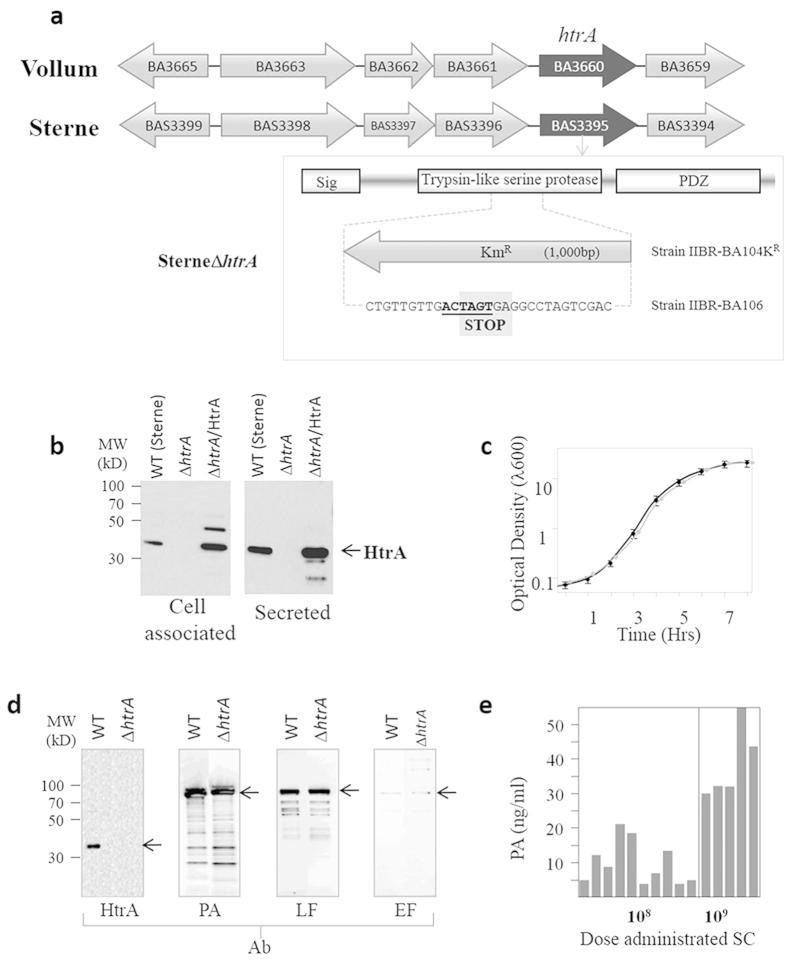
Generation and phenotypic characterization of the SterneΔ*htrA* strain. (**a**) The genomic loci of the *htrA* gene in *B. anthracis* Vollum and Sterne strains is shown. The functional domains of the *htrA* gene (The trypsin-like domain is necessary for the proteolytic activity of the protein, and the PDZ domain is necessary for the chaperone activity) and the signal peptide, which mediates export of the protein, are depicted in the boxed panel. Disruption of the *htrA* gene in the Sterne strain was achieved via two approaches: (i) introduction into the coding region of a kanamycin resistance gene (Km^R^) to generate the strain SterneΔ*htrA* IIBR-BA104K^R^; and (ii) marker-less replacement of the same coding region with the indicated DNA-sequence to generate the strain SterneΔ*htrA* IIBR-BA106. The DNA sequence disrupting the *htrA* gene in strain IIBR-BA106 is indicated, the novel SpeI restriction site present in this strain is indicated in bold and underlined, and the two stop codons in the sequence are boxed and marked STOP. (**b**) The Western blot analysis using the anti-HtrA antibodies of the secreted (Secreted) and membrane proteins (Cell associated) collected from cultures of the parental Sterne strain (WT), the SterneΔ*htrA* strain and the trans-complemented SterneΔ*htrA*/HtrA strain is shown and demonstrates the complete elimination of *htrA* expression in the mutated strain and its restoration in the trans-complemented strain. (**c**) The growth profiles in BHI media are shown. The black and grey curves describe the growth profiles of the parental Sterne and SterneΔ*htrA* strain, respectively, which was evaluated in 3 independent experiments. The error bars indicate SD. (**d**) The *in-vitro* production of the toxin components PA, LF and EF by the SterneΔ*htrA* and parental (WT) strains was demonstrated by Western blot analysis of proteins secreted in the NBY-CO_2_ cultures (18 hrs post-inoculation) of both strains using anti-HtrA, anti-PA, anti-LF and anti-EF antibodies, as indicated. (**e**) The PA levels in the serum of individual guinea pigs 3 days post-inoculation via the SC route with various doses of SterneΔ*htrA* spores was determined, as indicated. The analyses described in (**b–e**) employed the SterneΔ*htrA* IIBR-BA104K^R^ clone. The two SterneΔ*htrA* disrupted mutants, IIBR-BA104K^R^ and IIBR-BA106, were indistinguishable in all the comparative phenotypic studies.

We reasoned that elimination of the *htrA* gene may extend the safety of the Sterne vaccine by decreasing its virulence. Consequently, a novel Sterne Δ*htrA* strain was generated by disruption of the *htrA* locus (NCBI locus tag GBAS_3995). The chromosomal context of the *htrA* gene in the Sterne strain is identical to that in the Vollum strain with *htrA* encoded as a monocistronic gene (Fig. 2a). Therefore, the gene targeting manipulation for disruption of the gene in the Sterne strain was carried out by allelic-exchange approaches (Fig. 1a, see also Materials and Methods) based on the upstream and downstream target homology sequences, which are identical to those employed for the disruption of the *htrA* gene in the Vollum strain^15^. The disruption was confirmed by direct sequencing of the mutated *htrA* locus as well as by Western blot analysis, which demonstrated the complete abrogation of HtrA expression (Fig. 2b). In addition, we have also generated a transcomplemented strain, SterneΔ*htrA*/HtrA, by introducing an extrachromosomal HtrA expression plasmid into the mutated strain, which restored HtrA synthesis to levels similar to those observed in the parental Sterne strain (Fig. 2b).

No growth differences were observed between the Δ*htrA* strain and the parental Sterne strain cultures (Fig. 2c). Furthermore, the Sterne and the SterneΔ*htrA* strains were indistinguishable with respect to their ability to germinate and sporulate (Supplementary Fig. S1, online). The phenotype of the mutated strain was determined *in vitro* with respect to the characteristics and hallmarks previously determined for the *htrA*-mutated *B. anthracis* Vollum strain^15^ and other bacteria^21^,^22^,^23^,^24^. These phenotypic characterizations, which included growth under temperature and oxidative stress conditions as well as expansion in cultured macrophages, are documented in Supplementary Figs S2 and S3 (online). Compared to the parental strain, the SterneΔ*htrA* strain demonstrated a limited ability to grow at high temperature, an increased sensitivity to oxidative stress caused by hydrogen peroxide (Supplementary Fig. S2, online) and a delay (of more than 8 hours) in the J774-macrophage infection assay (Supplementary Fig. S3, online). All these phenotypic alterations could be restored to the parental-like levels by trans-complementation in strain SterneΔ*htrA*/HtrA, which demonstrates that these characteristics are directly related to the disruption of the *htrA* gene.

### Production of toxins by the SterneΔ*htrA* strain

Considering the fact that HtrA is a chaperone involved in the secretion machinery^25^,^26^, the efficacy of a live vaccine based on the SterneΔ*htrA* strain could be compromised by a decreased ability of the mutated strain to synthesize and/or to secrete the *B. anthracis* toxins PA, LF and EF. Direct evaluation by Western blot analysis of the toxin level clearly demonstrated that the mutation did not appear to affect the level of PA, LF or EF produced by the SterneΔ*htrA* mutant strain (Fig. 2d). Both of the toxic subunits of the exotoxins, LF and EF, were secreted by the mutated strain and appeared to be indistinguishable from those secreted by the parental strain in a macrophage-lysis assay, which probes the functional integrity of LF (Supplementary Fig. S4, online) and in an ATP-depletion assay, which probes the functional integrity of EF (Supplementary Fig. S4, online). Furthermore, substantial levels of PA could be detected in the circulation of guinea pigs 72 hrs following SC injection of spores from the SterneΔ*htrA* strain (Fig. 2e), suggesting that the administered spores could germinate and secrete the bacterial toxin *in vivo* in the host, a prerequisite for the use of the mutated strain as a vaccine (see next section and Supplementary Fig. S5, on line, for a comparison of the PA levels detected in the circulation of guinea pigs infected with the Δ*htrA* mutant and the parental Sterne strains).

### *In vivo* pathogenicity studies in animal models of anthrax

**Figure 3:**
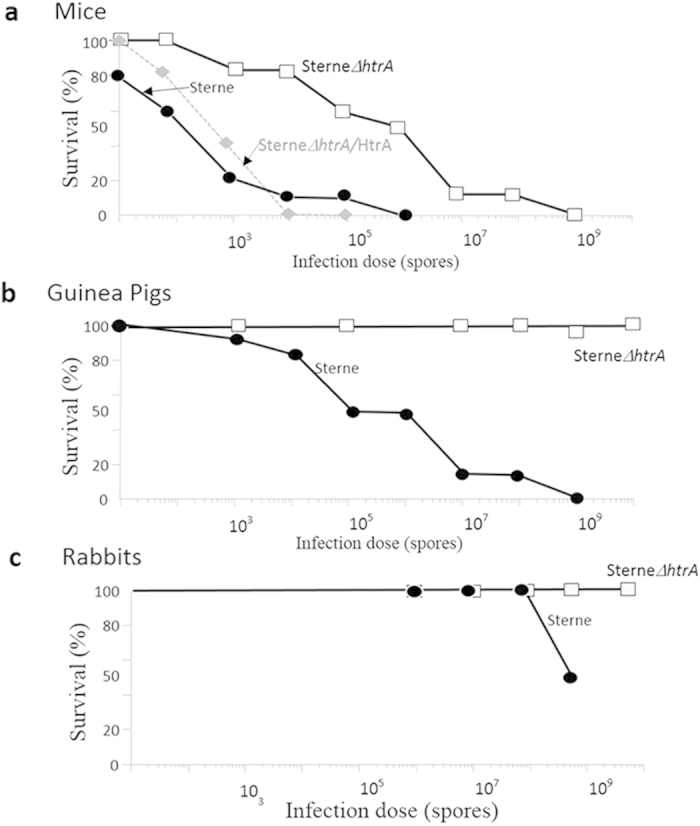
Survival profiles of mice, guinea pigs and rabbits infected subcutaneously with the Sterne and SterneΔ*htrA* strains. The animal groups (10 ICR mice, 20 guinea pigs or 5 rabbits per experimental group) were inoculated via the SC route with increasing spore doses of the parental Sterne strain (black circles), the mutated SterneΔ*htrA* strain (white squares) and the transcomplemented SterneΔ*htrA*/HtrA strain (tested in mice grey diamonds/grey dotted line). The survival of the animals was followed for 3 weeks. Table 2 shows the LD_50_ values calculated for the various strains in the three animal models. The experiments described employed the SterneΔ*htrA* IIBR-BA106 strain, but similar results were observed with the SterneΔ*htrA* IIBR-BA104K^R^ strain (not shown). In the guinea pig experiment, 19 of 20 animals survived a dose of 10^9^ SterneΔ*htrA* spores, but all the animals survived a dose of 10^10^ spores.

The impact of the *htrA* deletion on the virulence of the *B. anthracis* Sterne strain was evaluated in mice, guinea pigs and rabbits by SC administration of increasing doses of mutant spores in comparison to administration of Sterne parental-strain spores. The results depicted in Fig. 3 demonstrated that the virulence of the SterneΔ*htrA* bacteria was significantly attenuated (see Table 2 for the LD_50_ values of the mutated bacteria). The virulence attenuation index was almost 4 orders of magnitude in mice and guinea pigs. Furthermore, an increased dose-dependent mean-time to death (MTTD) was observed for the animals infected with the mutant SterneΔ*htrA* strain in the murine model. While for mice the MTTD for the Sterne parental strain was 4 days (for a dose of 10^5^ spores) and 2.5 days (for a dose of 10^8^ spores), in the case of the mutant SterneΔ*htrA* strain, the MTTD ranged from 8 days (for a dose of 10^5^ spores) to 3 days (for a dose of 10^8^ spores). Of note, the virulence attenuation could be alleviated by expression of HtrA in the transcomplemented strain SterneΔ*htrA*/HtrA, which was indistinguishable from the parental strain in the murine system (Fig. 3). In guinea pigs, 95% of the animals (19 out of 20) survived a dose of 10^9^ spores SterneΔ*htrA*, but 100% of animals (10 of 10) survived a higher dose of 10^10^. We therefore estimate the LD_50_ of SterneΔ*htrA* in guinea pigs to be greater than 10^10^. In rabbits, no animals succumbed upon administration of 5 × 10^9^ spores (higher doses were not attempted for practical reasons); therefore, the exact LD_50_ value could not be determined. (We note that the LD_50_ value of the parental Sterne strain in rabbits is approximately 5 × 10^8^ spores with occasional lethality at doses as low as 10^7^).

As high doses of the spores (e.g., 10^9^ spores/animal) could be administered to guinea pigs, relatively high serum levels of PA could be reached in infected animals 72 hours after SC infection with SterneΔ*htrA* (Supplementary Figure S5, online). Similar high levels could not be attained following vaccination with the parental Sterne due to the limit of tolerable doses for immunization (less than 10^7^ spores). The bacterial loads detected in the spleens of infected animals (Supplementary Figure S5, online) indicate that the mutated bacteria can migrate to the spleens of the guinea pigs. However, 32 days after SC administration, no bacteria could be detected in the spleens of animals infected with doses as high as 10^9^ spores.

### Immunization with SterneΔ*htrA* spores confers efficient protection against anthrax

**Figure 4:**
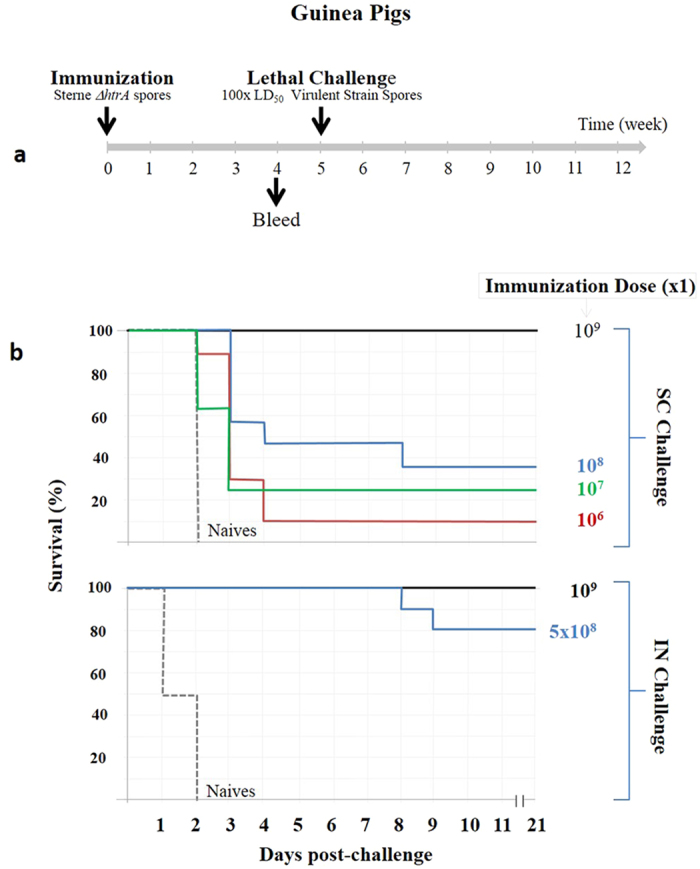
Protective immunity experiments in guinea pigs vaccinated by a single immunization with various doses of the SterneΔ*htrA* spores. (**a**) A Schematic representation of the single-immunization experiments is shown. (**b**) The Kaplan-Meier survival profiles of guinea pigs immunized with the indicated doses of SterneΔ*htrA* spores is shown. The animals (at least 10 per group) were challenged 5 weeks post-immunization with 100 × LD_50_ of fully virulent Vollum spores (pXO_1_^+^; pXO_2_^+^) by SC infection (10^4^ spores/animal, upper panel) or respiratory IN exposure (10^6^ spores/animal, lower panel). See Fig. 6 for the anti-PA, anti-bacterial antigens and neutralizing antibody titres measured in the pre-challenged blood samples. The experiments described in this and all subsequent figures employed the SterneΔ*htrA* IIBR-BA106 strain.

**Figure 5:**
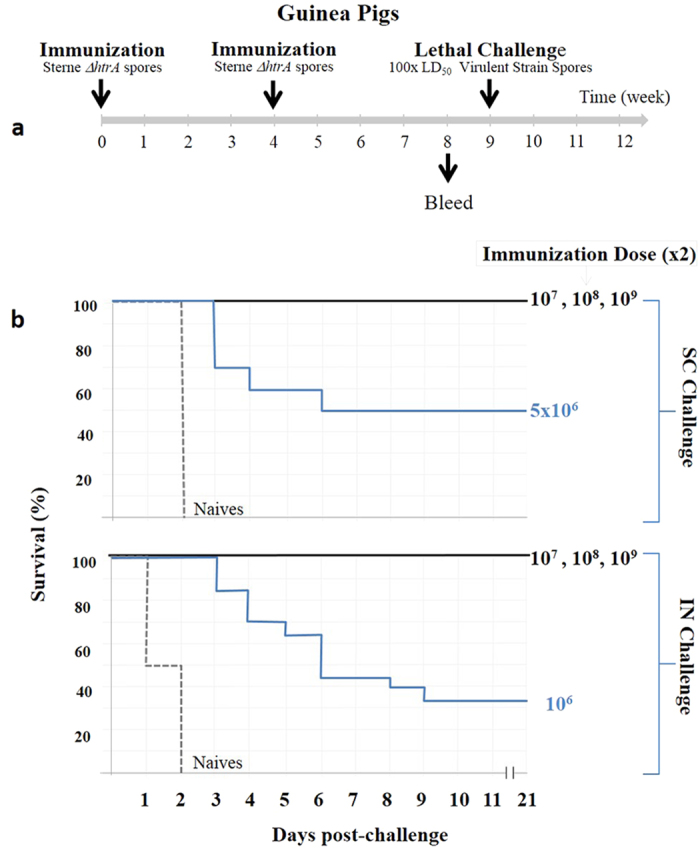
Protective immunity experiments in guinea pigs vaccinated with 2 immunizations of various SterneΔ*htrA* spore doses. (**a**) A schematic representation of the double-immunization experiments is shown. (**b**) The Kaplan-Meier survival profiles of the guinea pigs (at least 10 guinea pigs/group) immunized with the indicated doses of SterneΔ*htrA* spores is shown. The animals were challenged 5 weeks after the second immunization (boost) with 100 × LD_50_ of the fully virulent Vollum spores (pXO_1_^+^; pXO_2_^+^) by SC infection (10^4^ spores/animal, upper panel) or IN exposure (10^6^ spores/animal, lower panel). See Fig. 6 for the pre-challenge anti-PA, anti-bacterial antigens and neutralizing antibody titres.

**Figure 6:**
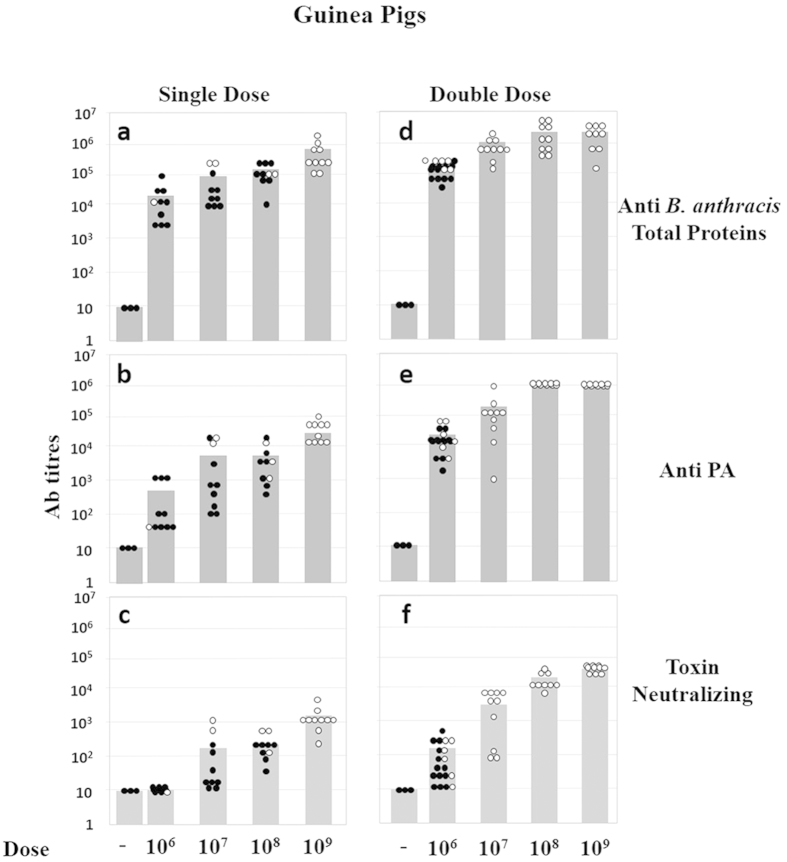
Antibody titres determined in guinea pigs 4 weeks after SC immunization with various SterneΔ*htrA* spore doses. Panels **(a–c**) Single-dose immunization data are shown (see also Fig. 4). Panels **(d–f**): Double-dose immunization data are shown (0 and 4 weeks; see also Fig. 5). Each dot represents the titre of one individual animal in the respective experimental group. The white and black dots indicate titres in individual animals that survived or did not survive the challenge, respectively. The grey histograms represent the GMT values of the indicated antibodies measured in the blood samples of each group. Panels (**a**,**d**): The titres of antibodies against total *B. anthracis* antigens other than those expressed from pXO1 or pXO2 (see material and methods) are shown; panels (**b**,**e)**: anti-PA antibody titres; panels (**c**,**f**): toxin neutralizing antibody titres.

To evaluate the potential use of the SterneΔ*htrA* strain as an efficient anthrax vaccine, both guinea pigs and rabbits were immunized with different doses, and their humoral immune response elicited by the immunization was evaluated. In addition, we evaluated the ability of the vaccinated animals to withstand a lethal challenge by a subcutaneous (SC) or respiratory (IN) exposure at a spore dose of 100 × LD_50_ of the fully virulent *B. anthracis* Vollum strain (see schematic description of the experiments in Figs 4a and 5a). The experiments in guinea pigs included SC administrations of a single immunization (10^6^–10^9^ spores/animal, Fig. 4) or two immunization doses 4-weeks apart (5 × 10^6^–10^9^ spores/animal, Fig. 5). Both immunization regimens elicited protective immune responses as depicted in Figs 4, 5, 6. Following the single immunization, we found that all vaccinated animals developed substantial titres of antibodies directed against total *B. anthracis* proteins (Fig. 6a) as well as antibodies against PA (Fig. 6b). These effects were dose-dependent. Significant titres of toxin neutralizing antibodies (TNA) could be detected in animals vaccinated with 10^7^ or more spores (Fig. 6c). In the double-immunization experimental groups, the humoral response was 1–2 orders of magnitude higher than that observed after a single immunization with the same dose (compare Fig. 6b–e and Fig. 6c–f). These observations underline the efficient boost effect promoted by a second dose of the vaccine spores administered 4 weeks after the initial immunization.

Vaccination of guinea pigs with the SterneΔ*htrA* strain could protect animals from a subsequent SC or IN challenge with 100 × LD_50_ of the fully virulent *B. anthracis* Vollum strain (Figs 4 and 5). We observed that 80% of the animals vaccinated once with 5 × 10^8^ spores survived IN challenge, and 100% of the animals vaccinated with 10^9^ spores were protected (Fig. 4). Full protection was also observed in animals vaccinated twice with more than 10^7^ spores (Fig. 5), a dose that was 3 orders of magnitude lower than the calculated LD_50_ of the SterneΔ*htrA* vaccine strain in guinea pigs ( > 10^10^, Fig. 3 and Table 2).

**Figure 7:**
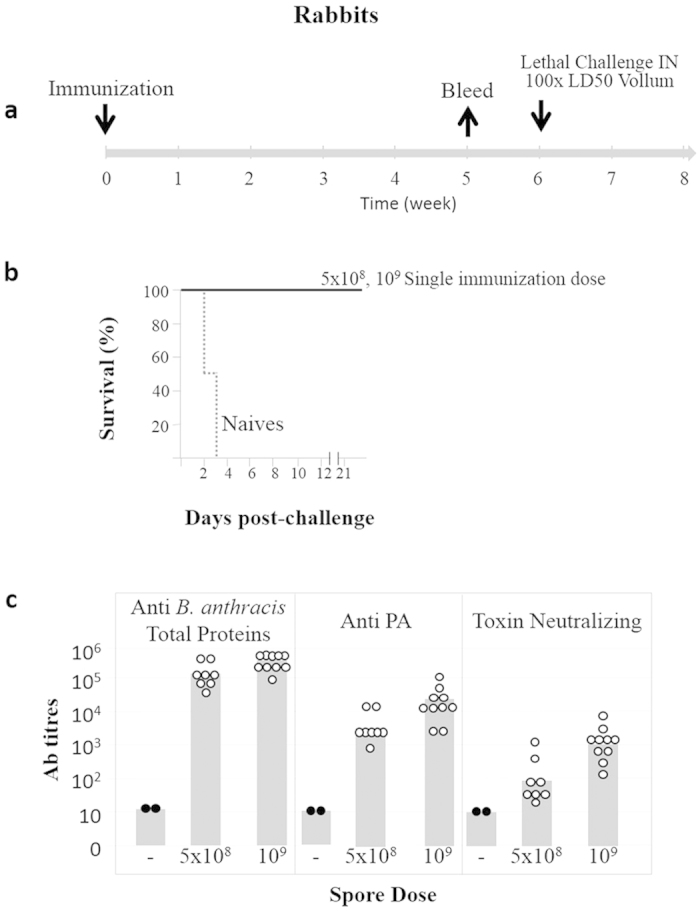
Protective immunity experiments in rabbits following a single immunization with various SterneΔ*htrA* spore doses. (**a**) A schematic representation of the immunization experiments is shown. (**b**): The Kaplan-Meyer survival profiles of rabbits immunized with the indicated doses of SterneΔ*htrA* spores is shown. The animals were challenged (5 weeks post-immunization) with 100 × LD_50_ of fully virulent Vollum spores (pXO_1_^+^; pXO_2_^+^) by IN exposure (10^6^ spores/animal). (**c**) The pre-challenge antibodies titres (against total-bacterial antigens, PA and anti-toxin neutralizing antibodies) were determined in guinea pigs (at least 8 animals per group) 4 weeks after immunization with the various doses of the SterneΔ*htrA* spores. The white and black dots indicate titres in individual animals that survived or did not survive the challenge, respectively. The grey histograms represent the GMT values of the indicated antibodies measured in the blood samples of each group.

The protective value of the vaccine was further confirmed in rabbits. We observed that administration of one dose of 5 × 10^8^ spores of the SterneΔ*htrA* strain resulted in a strong humoral anti-toxin and antibacterial response, which promoted full protection of animals in a subsequent IN challenge (Fig. 7). Again this immunization dose is at least 10-fold lower than the lowest possible LD_50_ dose of the SterneΔ*htrA* mutant (note that none of the rabbits died after exposure to the highest infection dose tested, see Table 2).

The longevity of the immune state was addressed in guinea pigs vaccinated with one dose (10^9^) or two doses (10^8^, 4 weeks apart) of SterneΔ*htrA* spores. In these experiments, the antibody titres of randomly selected animals from the various vaccination groups were determined at different time points following the last immunization. The data in Supplementary Table S1 (online) show that the humoral response declined gradually yet was maintained at a sufficiently high protective level in both immunization modes, and provided complete protection to all animals (20 animals/group) against an IN lethal challenge (100 × LD_50_) 52 weeks after vaccination.

The results presented above clearly demonstrate that disruption of the *htrA* gene can be implemented for the development of a safe live-attenuated anthrax vaccine without compromising the protective characteristic exhibited by the parental Sterne vaccine strain.

## Discussion

The currently licensed human anthrax vaccines contain filtered supernatants of avirulent *B. anthracis* strains potentiated by various adjuvants^3^,^16^. The active ingredient of these formulations is the toxin subunit PA. Although these vaccines were demonstrated as efficient and safe, they suffer from a cumbersome vaccination schedule that involves multiple initial administrations and yearly boost immunizations. Efforts to improve the sub-unit vaccine by bacterial additives or adjuvants^3^ are paralleled by the alternative approach, which relies on live attenuated strains as a basis for a next-generation vaccine. For example, several studies explored the possibility of using non-virulent mutated strains of *B. anthracis* as vaccines^27^,^28^,^29^ or even heterologous platforms, such as vaccinia virus, *Bacillus subtillis* or *Salmonella typhimurium*^30^,^31^,^32^,^33^, expressing recombinant forms of PA. In the past, we developed a nontoxinogenic and nonencapsulated recombinant spore vaccine based on a pXO1 and pXO2 cured variant of the nonproteolytic V770-NPI-R strain^34^,^35^,^36^. In the present study, we report on a novel live attenuated vaccine strain based on the existing Sterne veterinary vaccine strain (currently precluded from human use in the Western world, see Introduction) that is attenuated for virulence through deletion of the *htrA* gene. HtrA is a bifunctional chaperone and protease that was shown to be essential for the manifestation of *B. anthracis* virulence possibly owing to its role in protein quality control, which is necessary for overcoming the stress insults encountered by the bacteria in the host^15^,^37^. Here, we demonstrated in three animal models that extensive attenuation of the virulent Vollum strain, as well as that of the Sterne vaccine strain, can be achieved by disruption of *htrA* (Figs 1 and 3 and Table 2).

The success of a live attenuated vaccine encompasses a delicate balance between low virulence (hence amenable with human use) and the ability to promote a protective immune response. Virulence attenuation may lower the pathogenicity of the micro-organism to the extent that its interaction with the host results in an immune response insufficient to promote protection in a subsequent encounter with the fully virulent strain (a central theme in vaccine development^38^). The observations reported here clearly demonstrate that in spite of its markedly reduced virulence, the SterneΔ*htrA* strain elicits robust protection against a lethal challenge with the fully virulent Vollum strain administered via the SC or IN routes in vaccinated guinea pigs and rabbits (Figs 4, 5, 6, 7 and Supplementary Table S1, online). Furthermore, inspection of the anti-PA, anti-bacterial proteins and toxin-neutralizing antibody titres in vaccinated animals demonstrates that the immune response following vaccination with the Sterne Δ*htrA* strain (Figs 6 and 7 and Supplementary Table S1, online) targets both the toxin and other bacterial antigens. Therefore, this response addresses both toxaemia and the bacteremia, which are both associated with *B. anthracis* infection. Of note, full-protection of rabbits vaccinated with a PA sub-unit vaccine correlates with at least 3-fold higher levels of neutralizing antibodies^39^,^40^ compared to the antibodies in the fully protected animals following SterneΔ*htrA* vaccination. Similarly, in the guinea pig immunization experiments (Fig. 6), some animals exhibited very low levels of protective toxin neutralizing antibodies and yet were fully protected against a lethal challenge dose of 100 × LD_50_. These results suggest that the immune response elicited by additional antigens present in the live attenuated vaccine provides a significant added value to the protective anti-PA response. The observations reported here are in line with previous studies showing that vaccination of experimental animals with the Sterne strain is more efficient than the sub-unit PA vaccination^18^,^41^. Finally, the survival of all the guinea pigs immunized with a single dose of SterneΔ*htrA* after a lethal challenge at 52 weeks post-vaccination is a clear indication of the extensive potency and efficacy of this live attenuated vaccine (Supplementary Table S1, online).

**Figure 8:**
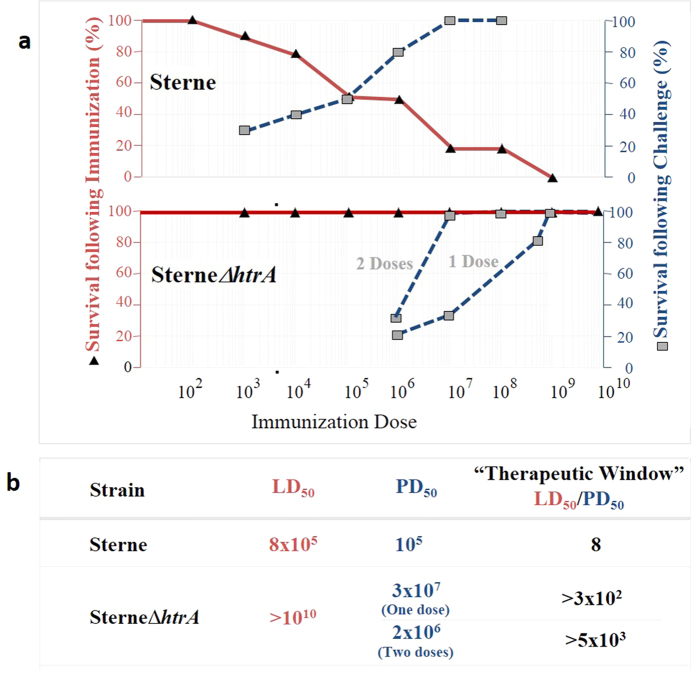
Extension of the therapeutic window of the live attenuated SterneΔ*htrA* spore vaccine compared to the Sterne parental vaccine strain in guinea pigs. This figure is based on results summarized in Table 2, Figs 4, 5, and 6 and on the Sterne vaccinated guinea pig protection data; a, upper panel: Sterne strain; lower panel: SterneΔ*htrA* strain. The red line (black triangles) represents the survival profile of guinea pigs immunized with increasing doses of spores as indicated on the left y-axis (red text). The blue dotted line represents the survival after challenge of the guinea pigs immunized with the indicated single or double spore doses. Note: (i) Not all the animals immunized with Sterne spores at doses lower than the LD_50_ survived the lethal challenge of Vollum, and 50% survival after such a challenge (Protective Dose 50%, PD_50_) is achieved by administration of a Sterne dose close to the LD_50_ value of the strain. (ii) Unlike the Sterne vaccine, 100% survival can be achieved with the SterneΔ*htrA* vaccine strain at a vaccination dose much lower than the LD_50_ value of the Sterne Δ*htrA* vaccine. Panel b: The calculated “therapeutic window” was determined from the ratio between the safety (LD_50_) and the efficacy (PD_50_) values of the two vaccine strains. Note that for SterneΔ*htrA*, only a lower limit of the LD_50_ was determined (Fig. 3 and Table 2), and therefore, the therapeutic window may be even wider than has been estimated in this figure.

The observations discussed above indicate that the novel SterneΔ*htrA* vaccine exhibits (i) significantly improved safety as manifested by its dramatic virulence attenuation, and (ii) high efficacy in protecting against infection by a fully virulent *B. anthracis* strain. The balance between these two parameters determines the value of the live attenuated vaccine, which may be quantified and fully appreciated by determining the “therapeutic window” of the vaccine in guinea pigs. This is schematically visualized by comparing the LD_50_ and the PD_50_ (Protective Dose 50%) as exhibited in guinea pigs by the parental Sterne strain and the novel SterneΔ*htrA* strain (Fig. 8). The conclusion from this presentation is that in the case of the Sterne vaccine, survival from a lethal challenge can be achieved after immunization at doses that are close to the LD_50_ value (thus with a minimal “therapeutic window” ratio of LD_50_ to PD_50_ of only 8.0). However, in the case of the SterneΔ*htrA* vaccine, administration of as little as 10^−2^ × LD_50_ (for single dose immunization) or even 10^−3^ × LD_50_ (for the double-dose immunization) is sufficient to promote over 50% survival after challenge (Fig. 8). This calculation estimates that the “therapeutic window” of the novel SterneΔ*htrA* is at least two orders of magnitude higher compared to that of the parental Sterne veterinary vaccine, which supports the conclusion that the former is a substantially safer vaccine.

In conclusion, the immunization of guinea pigs and rabbits by a single or double administration of this novel SterneΔ*htrA* live-attenuated spore vaccine at doses well below the LD_50_ of the vaccine strain elicits full protection against subcutaneous or airway infection of a fully virulent anthrax strain. Therefore, the SterneΔ*htrA* strain can serve as an appropriate platform for a next-generation human anthrax vaccine.

## Materials and Methods

### Bacterial strains, media, growth conditions and stress treatments

The *B. anthracis* strains used in this study are listed in the online Supplementary Table S3. The cells were cultured either in FAG media^34^, Brain-Heart Infusion (BHI, DIFCO/Becton Dickinson, MD, USA) or NBY media (0.8% [w/vol] Nutrient Broth [Difco], 0.3% Yeast extract [Difco] and 0.5% Glucose) at 37 °C with vigorous agitation. For the identification of CO_2_-induced proteins, the cells were grown at 37 °C in NBY supplemented with 0.9% NaHCO_3_ in hermetically sealed flasks without agitation (referred hereafter as NBY-CO_2_). NBY-CO_2_ media promotes efficient toxin production^9^,^14^,^42^. The spores were prepared in Schaeffer’s sporulation media (SSM) at 34 °C for 72 hours with vigorous shaking. In all the cases, the cultures were initiated with a starter inoculum diluted to a final OD (660 nm) of 0.1. For the H_2_O_2_ stress test, the cells were allowed to grow under optimal conditions for 2.5–3 hours (typically the time needed to enter logarithmic phase) and were then split into BHI cultures containing the indicated final concentrations of H_2_O_2_. The growth rate μ (expressed in hr^−1^) was calculated as 60 × [ln(OD_2_/OD_1_)]/(t_2_–t_1_), where OD_1_ and OD_2_ are the optical densities (at 600 nm) measured at two time-points (t_1_ and t_2_ expressed in minutes) along the logarithmic growth phase.

*Escherichia coli* strains (Supplementary Table S2, online) were used for plasmid construction. The antibiotic concentrations used for selection in Luria-Bertani (LB, Difco) agar/broth were as follows: for *E. coli* strains, ampicillin (Ap, 100 μg ml^−1^); for *B. anthracis* strains, kanamycin (Km, 10 μg ml^−1^), chloramphenicol (Cm, 7.5 μg ml^−1^) and erythromycin (Em, 5 μg ml^−1^).

### Plasmid and strain construction

The plasmids and oligonucleotide primers used in this study are summarized in Supplementary Table S2 (online). The oligonucleotide primers were designed according to the genomic sequence of *B. anthracis* “Ames ancestor” strain (accession number AE017334, GI 50082967) and were prepared using the Expedite synthesizer (Applied Biosystems). The sequence identity of the *htrA* locus in the Vollum and Sterne strains was verified by whole-genome sequencing. Genomic DNA was extracted as previously described^42^,^43^. The PCR amplifications were performed using the *Taq* (Qiagen) or Expand High Fidelity (Roche) systems. The DNA sequences were determined by the ABI rhodamine termination reaction (ABI310 Genetic Analyzer, Applied Biosystems). The plasmid used for generation of the *htrA*-disrupted strains and the trans-complementation plasmids for extrachromosomal HtrA expression were previously described^15^. All the plasmids transformed into the Vollum or Sterne strains were first propagated in the methylation-deficient *E. coli* strain GM2929. *B. anthracis* cells were electro-transformed as previously described^34^. To disrupt the *htr*A gene by one-step homologous recombination, an allelic exchange technique was performed as follows: plasmid pEO-*htr*A was introduced into competent cells of the Sterne strain, and the transformants were selected for Km^R^ at 30 °C. The integrants were recovered by growing the transformants in LB broth at 30 °C for 1.5 h, shifting to 38 °C (non-permissive temperature) for 6 h, plating serial dilutions on LB plates containing Km and were then incubated at 38 °C for 12–16 h. Single colonies were selected, resuspended in 0.1 ml LB broth, spotted (5 μl) on duplicate LB plates containing Km or Em, and then the twin plates were incubated either at 42 °C or 30 °C for 12–16 h. The deletion mutants were isolated as Km^R^ Em^S^ clones that failed to grow at 42 °C (as expected from the phenotype associated with *htrA*-gene disruption). The deletion of the internal fragment of *htr*A and insertion of the Km^R^ cassette into the chromosome were confirmed by PCR using flanking chromosomal primers and primers derived from the Km cassette (primers used: HTRA9/KANA2C and KANA1/HTRA12C for the upstream and downstream integration *htr*A/Km junctions, respectively) and by sequencing of the *htrA* locus. The above approach generated a SterneΔ*htrA* strain that carries kanamycin resistance (IIBR collection BA104K^R^). A two-step gene-disruption technique was implemented for the generation of an additional *htrA*-disrupted strain devoid of antibiotic resistance. This technique, which is based on the activity of the I-SceI recombinase, was previously reported^44^. The *B. anthracis* cells are first transfected with the plasmid pEGS-*htrA*, which contains the same upstream and downstream *htrA* gene homology regions as the plasmid pEO-*htr*A, an I SceI recombinase recognition site at the 5’ of the downstream *htrA* homology region, an erythromycin resistance gene and a GFPuv gene^45^,^46^. The transfection results in the generation of a strain exhibiting a single cross-over integrant that is further resolved to the desired double cross-over markerless disrupted strain by expression of the I SceI recombinase (from the transfected pXX-I SceI plasmid). The above marker-less allelic exchange approach generated a SterneΔ*htrA* strain devoid of antibiotic resistance (designated in the IIBR collection BA106). The two SterneΔ*htrA* strains, BA104K^R^ and BA106, were phenotypically indistinguishable. Throughout this report, the strain used for the various experiments is indicated in the relevant figure legend (see Fig. 2a for a schematic description of the disrupted *htrA* gene). Complementation was accomplished by transforming pASCHtrA into the *htr*A mutant strain.

### SDS-PAGE, Western blot analyses, antibodies, and ELISA

SDS-PAGE was carried out on 4–12% NuPAGE^®^ Bis-Tris gels (Invitrogen). For comparisons between proteins secreted by different strains, equal amounts of protein (50 μg) from the corresponding strains were loaded side by side on SDS-PAGE. The Western blots were generated using the Nitrocellulose Western iBlot Gel Transfer Semi-dry system (Invitrogen). Visualization of the immunoreactive bands was accomplished using an ECL (electrochemiluminescence) reaction (Pierce SuperSignal^®^ West Pico Chemiluminescent substrate kit, Thermo Scientific) mediated by peroxidase-conjugated secondary antibodies (Amersham) detected by the FUJIFILM LAS-3000 detection system. The following antibodies were used in this study: (i) Specific anti-HtrA antibodies, which were obtained by DNA immunization with the pCI-htrA plasmid in female outbred ICR mice using the Helios Gene Gun System (Bio-Rad) as previously described^47^; (ii) Rabbit anti-PA antibodies^35^,^48^; (iii) Anti-LF and (iv) anti-EF mouse antibodies^34^,^36^. The primary and secondary antibodies were used at 1:1000 and 1:5000 dilutions, respectively.

The ELISA tests for the quantification of PA in sera samples and the detection of anti-PA antibodies in the serum of infected animals were carried out as previously described^34^,^36^,^48^,^49^,^50^.

The ELISAs for the quantification of anti-bacterial antibody titres in the circulation of vaccinated animals were carried out on microtitre plates coated with a *B. anthracis* urea-extracted proteinous preparation^8^ as previously described^50^. The proteinous extracts were prepared from a nonvirulent, non-toxinogenic and non-encapsulated ΔVollum strain (pXO1^−^, pXO2^−^). Therefore, the ELISA test monitors the humoral response elicited against *B. anthracis* chromosomally encoded exposed proteins and does not include the anti-toxin response nor the capsular antigens (any antigen encoded by pXO1 or pXO2 is absent).

Quantification of the toxin-neutralizing antibody (TNA) titres in the circulation of vaccinated animals was carried out by a J774A.1 macrophage-lysis inhibition assay in the presence of excess PA and LF as previously described^36^.

### Dilution drop assays

Ten-fold serial dilutions were generated from logarithmic growth phase bacteria (0.5 OD_600_) and were dropped (50 μl) in duplicate on LB agar plates that were incubated overnight at 37 °C or 42 °C.

### LF and EF functional assay

Bacteria (0.1 OD, 600 nm) were used to inoculate 1 ml cultures of DMEM (Biological Industries, Bet Haemek, Israel) supplemented with 10% FCS in 24-well microtitre plates and were incubated for 16 hrs at 37 °C in a 10% CO_2_ atmosphere. The culture supernatant was collected by centrifugation (5 min; 4,000 × g) and was filtered twice through a 0.22-μm filter. LF activity in the culture was assayed by layering the two-fold dilutions of the filtered supernatant in the presence of 0.25 μg/ml PA on sub-confluent monolayers of J774A.1 cells (plated in 96-well microtitre plates at a density of 5 × 10^4^ cells/ml one day prior to assay). The cells were incubated for approximately 4 hrs at 37 °C under 5% CO_2_ until the cytopathic effect was observed microscopically. Visualization of the cell lysis was achieved by addition of XTT-reagent (Cell Proliferation Kit, Biological Industries, Bet Haemek, Israel), and the lysis was quantified using a spectrophotometer (Novaspec Plus, Amersham). A luminescent ATP-depletion EF functional assay based on the calmodulin-dependent adenylate cyclase activity of EF was developed. This assay is based on the strict dependence of a luminescent luciferase reaction on ATP, which can be efficiently converted to cAMP by adenylate cyclases. In brief, the DMEM culture supernatants (as above) were concentrated 5 times using the Microcon concentration/buffer exchange system (Millipore YM-10, Merck, Ireland). For the ATP depletion, 10 μl of the concentrated supernatant (containing 3–5 ng of EF as quantified by ELISA) was added to 50 μl of the luciferase assay reagent (Promega, Madison, WI, USA) containing 1 mM Calmodulin (Sigma) and 0.5 mM CaCl_2_ (Merck) and was incubated in a polysorp fluoroNunc microtitre plate (Nunc) at 30 °C for 2.5 hrs. QuantiLum recombinant luciferase (Promega) was added to the reaction mixture, and the luminescence was measured immediately with a luminometer (VICTOR^3^TM, Perkin Elmer).

### Evaluation of sporulation and germination efficiency

To determine the sporulation efficiency, bacteria were sampled from cultures grown in SSM (at 34 °C with shaking at 200 rpm), and the percentage of spores was determined by viable plating before and after a 20 min heat incubation step (70 °C). While both vegetative cells and spores are equally efficient in generating colonies, after heat incubation only the heat-resistant spores are able to generate colonies. The spore percentage was calculated as the ratio between the CFU count after heat treatment divided by the CFU count before heat treatment (x 100). The germination efficiency was determined in BHI media inoculated with heat-treated spores at a final OD_600_ of 0.5 by spectrophotometrically following the decrease in the optical absorbance at 600 nm, which is characteristic of spore depletion in the culture (Alvarez *et al*., 2010). The percentage of spores was calculated by comparing the optical absorbance before ( = 100% spores) and at the various time points after the initiation of germination. The results were confirmed by viable counting before and after a heat treatment step (70 °C, 20 min).

### Macrophage infection assay

The macrophage infections were carried out as previously described^43^,^49^. In brief, J774.1 murine macrophages grown in DMEM supplemented with 10% foetal calf serum (37 °C; 5% CO_2_ atmosphere) were seeded at a concentration of 10^5^ cells/well in a 24-wells plate one day prior to infection. The cells were incubated for 1 hour in the presence of 5 × 10^5^ *B. anthracis* spores/well, were washed extensively with DMEM supplemented with 2.5 μg/ml^−1^ gentamycin, were incubated for 3 hrs in the presence of 2.5 μg/ml gentamycin and were washed again and layered with fresh media. Under these conditions, essentially all the bacteria that are not internalized are removed (as tested by viable bacterial counts). The supernatants from identically treated wells were harvested at various time points, and the viable bacterial counts were determined. Macrophage lysis as a consequence of the multiplication of the bacteria was monitored by assaying the accumulated lactate dehydrogenase (LDH) released in the media from the damaged cells at the harvesting times using a standard LDH-L kit (Thermo-Fisher Scientific, Middletown, VA, USA).

### Dissemination of bacteria to spleens of infected guinea pigs

Female Hartley guinea pigs (Charles River Laboratories), weighting 220 to 250 g were infected SC with various doses of Sterne or Sterne Δ*htrA* spores. At various time points (1–35 days) post-infection three randomly selected animals were euthanized and their spleens were removed, homogenized and plated in 10-fold serial dilutions for determining the total bacterial load. Similar samples were plated after heat-shock (70 °C, 20 minutes) to determine the percentage of spores in the organs.

### Infection of experimental animals

Female Hartley guinea pigs (220 to 250 g, Charles River Laboratories), ICR mice (20–25 g, Harlan) and NZ rabbits (2.5 kg, Charles River Laboratories) were infected with various spore preparations. Prior to infection, the spore preparations were heat-shocked (70 °C, 20 min) to kill the residual vegetative bacteria and were serially diluted 10-fold in saline to produce spore suspensions within the range 10^2^–10^11^ per ml. A 0.1 ml spore dose volume was administered subcutaneously (SC) to each guinea pig or mouse. A total of 5–10 guinea pigs per dose-strain were used. The rabbits were injected subcutaneously with 1 ml of PBS containing the desired amount of spores. The remaining spore dose suspensions were plated for total viable counts (CFU ml^−1^) to confirm the dose administered to the animals. The attenuation index is defined as the ratio between the LD_50_ exhibited by the mutated strain and the WT parental ancestor. At seventy-two hours after infection, some of the guinea pigs were bled by cardiac puncture to determine the levels of PA in the peripheral circulation. The animals were observed daily for 21 days. Four weeks post-infection, the surviving guinea pigs or rabbits were bled by cardiac puncture or from the ear vein, respectively, for serological studies. In the guinea pig and rabbit immunization experiments, at 5 weeks post-infection (or 52 weeks in the immune longevity experiments) the animals were challenged SC or IN with the indicated lethal dose of the parental *B. anthracis* Vollum strain (SC-LD_50_ = approximately 100 spores, IN-LD_50_ = approximately 10^4^ spores). The IN infection was performed following anaesthesia of the guinea pigs or rabbits by nasal instillation of 100 μl PBS (guinea pigs) or 500 μl PBS (rabbits) containing the desired dose (guinea pigs: 50 μl/nostril; rabbits 250 μl/nostril). The spore lethal dose required to kill 50% (LD_50_) of the animals was calculated by non-linear fit regression using the GraphPad Prism (version 5) statistical analysis software (San Diego, CA).

### Longevity of the immunological status in vaccinated guinea pigs

Anti-PA, anti-bacteria and toxin-neutralizing (TNA) titres in the blood samples of vaccinated guinea pigs were determined at various time points post-immunization. The blood samples were collected from 5 randomly selected animals from each group, and their antibody titres were determined. Therefore, the titre values (in all time-points except the last time-point preceding challenge, Supplementary Table S1, online) represent the response of 5 different animals from the same experimental group. At fifty weeks post-immunization, blood samples were collected from *all* animals for the evaluation of antibody titres. At fifty-two weeks post-immunization, all the animals were challenged with 100 × LD_50_ of the fully virulent *B. anthracis* Vollum strain by IN infection. Unlike the vaccinated animals, all five naïve control animals (kept in similar conditions for the same 52 week time period) succumbed to the 100 × LD_50_ IN challenge.

### Statistical analysis

Kaplan-Meier analyses and dose-dependent survival analyses were used for survival evaluation. The survival data were analysed using GraphPad Prism (version 5) statistical analysis software. A t-test was used to compare the mean survival time between the groups. A two-tailed log rank test was used to determine the statistical significance of differences between groups. A p value of <0.05 was considered statistically significant.

All the animal experiments were approved by the IIBR committee for animal research. The experimental animals were handled according to the National Research Council 1996 Guide for the Care and Use of Laboratory Animals and regulations of the IIBR Animal Use Committee.

## Additional Information

**How to cite this article**: Chitlaru, T. *et al*. Next-Generation *Bacillus anthracis* Live Attenuated Spore Vaccine Based on the *htrA*^-^ (High Temperature Requirement A) Sterne Strain. *Sci. Rep.* **6**, 18908; doi: 10.1038/srep18908 (2016).

## Supplementary Material

Supplementary Information

## Figures and Tables

**Table 1:** Examples of reported *B. anthracis* virulence attenuation values associated with disruption of genes not associated with classical virulence factors (see text).

**Gene(s) disrupted**	**Function**	**Parental strain**	**Animal model**	**Infection route**	**Attenuation Index (LD**_50_Δ**/LD**_50_**WT)**	**Reference**
*aro*	Aromatic acid synthesis	Sterne	Mouse	*SC*	>10,000	27
*nos*	Oxidative Stress	Sterne	Mouse	*SC*	1,000	51
*sod15, sodA1, sodC, sodA2*	Superoxide detoxification	Sterne	Mouse	*IN*	100	52
*clpX*	Proteolytic quality control	Sterne Sterne Ames	Mouse Mouse GP Mouse	*SC IN IP IP*	10–100 10–100 1,000 1	53
*purH*	Purine biosynthesis	Ames	Mouse Rabbit GP	*IN SC IM*	1 1 1,000	54
*bslA*	Adhesion	Ames	GP	*SC*	200	55
		Vollum	GP	*SC*	1	12
*mntA*	Mn(II) transport	Vollum	GP	*SC IN*	10,000 1	43
*isdC isdJ isdK*	Acquisition of iron	Vollum	GP	*SC*	1	42
*codY*	Transcription regulator	9602	Mouse GP	*SC*	20 10,000	56
*htrA*	Stress tolerance, proteolysis	Vollum	GP GP	*SC IN*	3,000,000 > 10,000	15

**Table 2:** LD_50_ values of various *B. anthracis* strains and their respective Δ*htrA* mutants (SC administration).

	**B. anthracis strain**			
	**Vollum (pXO_1_^+^/pXO_2_^+^)**		**Sterne (pXO_1_^+^/pXO_2_^−^)**	
	WT	ΔhtrA	WT	ΔhtrA
Animal Model Mice (ICR)	10	3 × 10^5^	1.6 × 10^2^	6 × 10^5^
Guinea Pigs	50–100	3 × 10^8^	8 × 10^5^	>10^10^
Rabbits	50–100	>5 × 10^8^	5 × 10^8^	>5 × 10^9^

## References

[b1] Driks A. The Bacillus anthracis spore. Mol Aspects Med 30, 368–373 (2009). 19683018 10.1016/j.mam.2009.08.001

[b2] Friedlander A. M. . Postexposure prophylaxis against experimental inhalation anthrax. J Infect Dis 167, 1239–1243 (1993). 8486963 10.1093/infdis/167.5.1239

[b3] Chitlaru T., Altboum Z., Reuveny S. & Shafferman A. Progress and novel strategies in vaccine development and treatment of anthrax. Immunol Rev 239, 221–236 (2011). 21198675 10.1111/j.1600-065X.2010.00969.x

[b4] Koehler T. M. Bacillus anthracis physiology and genetics. Mol Aspects Med 30, 386–396 (2009). 19654018 10.1016/j.mam.2009.07.004 PMC2784286

[b5] Ariel N. . Search for potential vaccine candidate open reading frames in the Bacillus anthracis virulence plasmid pXO1: in silico and *in vitro* screening. Infect Immun 70, 6817–6827 (2002). 12438358 10.1128/IAI.70.12.6817-6827.2002 PMC133087

[b6] Ariel N. . Genome-based bioinformatic selection of chromosomal Bacillus anthracis putative vaccine candidates coupled with proteomic identification of surface-associated antigens. Infect Immun 71, 4563–4579 (2003). 12874336 10.1128/IAI.71.8.4563-4579.2003 PMC165985

[b7] Chitlaru T. & Shafferman A. Proteomic studies of Bacillus anthracis. Future Microbiol 4, 983–998 (2009). 19824790 10.2217/fmb.09.73

[b8] Chitlaru T. . Identification of chromosomally encoded membranal polypeptides of Bacillus anthracis by a proteomic analysis: prevalence of proteins containing S-layer homology domains. Proteomics 4, 677–691 (2004). 14997491 10.1002/pmic.200300575

[b9] Chitlaru T., Gat O., Gozlan Y., Ariel N. & Shafferman A. Differential proteomic analysis of the Bacillus anthracis secretome: distinct plasmid and chromosome CO2-dependent cross talk mechanisms modulate extracellular proteolytic activities. J Bacteriol 188, 3551–3571 (2006). 16672610 10.1128/JB.188.10.3551-3571.2006 PMC1482852

[b10] Chitlaru T., Gat O., Grosfeld H., Inbar I., Gozlan Y. & Shafferman A. Identification of *in vivo*-expressed immunogenic proteins by serological proteome analysis of the Bacillus anthracis secretome. Infect Immun 75, 2841–2852 (2007). 17353282 10.1128/IAI.02029-06 PMC1932864

[b11] Gat O. . Search for Bacillus anthracis potential vaccine candidates by a functional genomic-serologic screen. Infect Immun 74, 3987–4001 (2006). 16790772 10.1128/IAI.00174-06 PMC1489694

[b12] Shafferman A. . Reverse Vaccinology in Bacillus anthracis Bacillus anthracis. In: The Challenge of Highly Pathogenic Microorganisms (ed^(eds Shafferman A., Ordentlich A., Velan B. ). Springer: Netherlands, (2010).

[b13] Sela-Abramovich S., Chitlaru T., Gat O., Grosfeld H., Cohen O. & Shafferman A. Novel and unique diagnostic biomarkers for Bacillus anthracis infection. Appl Environ Microbiol 75, 6157–6167 (2009). 19648366 10.1128/AEM.00766-09 PMC2753070

[b14] Chitlaru T., . Proteomic Studies of Bacillus anthracis Reveal *In Vitro* CO2-Modulation and Expression During Infection of Extracellular Proteases. In: The Challenge of Highly Pathogenic Microorganisms (eds Shafferman A., Ordentlich A., Velan B. ). Springer: Netherlands, (2010).

[b15] Chitlaru T., Zaide G., Ehrlich S., Inbar I., Cohen O. & Shafferman A. HtrA is a major virulence determinant of Bacillus anthracis. Mol Microbiol 81, 1542–1559 (2011). 21801240 10.1111/j.1365-2958.2011.07790.x

[b16] Friedlander A. M. & Little S. F. Advances in the development of next-generation anthrax vaccines. Vaccine 27 Suppl 4, D28–32 (2009). 19837282 10.1016/j.vaccine.2009.08.102

[b17] Shlyakhov E. N. & Rubinstein E. Human live anthrax vaccine in the former USSR. Vaccine 12, 727–730 (1994). 8091851 10.1016/0264-410x(94)90223-2

[b18] Little S. F. & Knudson G. B. Comparative efficacy of Bacillus anthracis live spore vaccine and protective antigen vaccine against anthrax in the guinea pig. Infect Immun 52, 509–512 (1986). 3084385 10.1128/iai.52.2.509-512.1986 PMC261029

[b19] Welkos S. L., Keener T. J. & Gibbs P. H. Differences in susceptibility of inbred mice to Bacillus anthracis. Infect Immun 51, 795–800 (1986). 3081444 10.1128/iai.51.3.795-800.1986 PMC260968

[b20] Goossens P. L. Animal models of human anthrax: the Quest for the Holy Grail. Mol Aspects Med 30, 467–480 (2009). 19665473 10.1016/j.mam.2009.07.005

[b21] Rigoulay C. . Comparative analysis of the roles of HtrA-like surface proteases in two virulent Staphylococcus aureus strains. Infect Immun 73, 563–572 (2005). 15618196 10.1128/IAI.73.1.563-572.2005 PMC538960

[b22] Pallen M. J. & Wren B. W. The HtrA family of serine proteases. Mol Microbiol 26, 209–221 (1997). 9383148 10.1046/j.1365-2958.1997.5601928.x

[b23] Biswas S. & Biswas I. Role of HtrA in surface protein expression and biofilm formation by Streptococcus mutans. Infect Immun 73, 6923–6934 (2005). 16177372 10.1128/IAI.73.10.6923-6934.2005 PMC1230926

[b24] Cortes G., de Astorza B., Benedi V. J. & Alberti S. Role of the htrA gene in Klebsiella pneumoniae virulence. Infect Immun 70, 4772–4776 (2002). 12183518 10.1128/IAI.70.9.4772-4776.2002 PMC128236

[b25] Antelmann H. . The extracellular proteome of Bacillus subtilis under secretion stress conditions. Mol Microbiol 49, 143–156 (2003). 12823817 10.1046/j.1365-2958.2003.03565.x

[b26] Clausen T., Kaiser M., Huber R. & Ehrmann M. HTRA proteases: regulated proteolysis in protein quality control. Nat Rev Mol Cell Biol 12, 152–162 (2011). 21326199 10.1038/nrm3065

[b27] Ivins B. E., Welkos S. L., Knudson G. B. & Little S. F. Immunization against anthrax with aromatic compound-dependent (Aro-) mutants of Bacillus anthracis and with recombinant strains of Bacillus subtilis that produce anthrax protective antigen. Infect Immun 58, 303–308 (1990). 2105269 10.1128/iai.58.2.303-308.1990 PMC258455

[b28] Pezard C., Weber M., Sirard J. C., Berche P. & Mock M. Protective immunity induced by Bacillus anthracis toxin-deficient strains. Infect Immun 63, 1369–1372 (1995). 7890396 10.1128/iai.63.4.1369-1372.1995 PMC173160

[b29] Barnard J. P. & Friedlander A. M. Vaccination against anthrax with attenuated recombinant strains of Bacillus anthracis that produce protective antigen. Infect Immun 67, 562–567 (1999). 9916059 10.1128/iai.67.2.562-567.1999 PMC96355

[b30] Coulson N. M., Fulop M. & Titball R. W. Bacillus anthracis protective antigen, expressed in Salmonella typhimurium SL 3261, affords protection against anthrax spore challenge. Vaccine 12, 1395–1401 (1994). 7887017 10.1016/0264-410x(94)90148-1

[b31] Iacono-Connors L. C., Welkos S. L., Ivins B. E. & Dalrymple J. M. Protection against anthrax with recombinant virus-expressed protective antigen in experimental animals. Infect Immun 59, 1961–1965 (1991). 1903769 10.1128/iai.59.6.1961-1965.1991 PMC257950

[b32] Ivins B. E. & Welkos S. L. Cloning and expression of the Bacillus anthracis protective antigen gene in Bacillus subtilis. Infect Immun 54, 537–542 (1986). 3021632 10.1128/iai.54.2.537-542.1986 PMC260194

[b33] Welkos S. L. & Friedlander A. M. Comparative safety and efficacy against Bacillus anthracis of protective antigen and live vaccines in mice. Microb Pathog 5, 127–139 (1988). 3148815 10.1016/0882-4010(88)90015-0

[b34] Cohen S. . Attenuated nontoxinogenic and nonencapsulated recombinant Bacillus anthracis spore vaccines protect against anthrax. Infect Immun 68, 4549–4558 (2000). 10899854 10.1128/iai.68.8.4549-4558.2000 PMC98371

[b35] Gat O. . Use of a promoter trap system in Bacillus anthracis and Bacillus subtilis for the development of recombinant protective antigen-based vaccines. Infect Immun 71, 801–813 (2003). 12540560 10.1128/IAI.71.2.801-813.2003 PMC145393

[b36] Mendelson I. . Efficacious, nontoxigenic Bacillus anthracis spore vaccines based on strains expressing mutant variants of lethal toxin components. Vaccine 23, 5688–5697 (2005). 16039760 10.1016/j.vaccine.2004.11.077

[b37] Skorko-Glonek J. . HtrA protease family as therapeutic targets. Curr Pharm Des 19, 977–1009 (2013). 23016688 10.2174/1381612811319060003

[b38] Galen J. E. & Curtiss R. 3rd. The delicate balance in genetically engineering live vaccines. Vaccine 32, 4376–4385 (2014). 24370705 10.1016/j.vaccine.2013.12.026 PMC4069233

[b39] Little S. F. . Duration of protection of rabbits after vaccination with Bacillus anthracis recombinant protective antigen vaccine. Vaccine 24, 2530–2536 (2006). 16417950 10.1016/j.vaccine.2005.12.028

[b40] Chawla A., Midha S. & Bhatnagar R. Efficacy of recombinant anthrax vaccine against Bacillus anthracis aerosol spore challenge: preclinical evaluation in rabbits and Rhesus monkeys. Biotechnol J 4, 391–399 (2009). 19296443 10.1002/biot.200800213

[b41] Ivins B. E. & Welkos S. L. Recent advances in the development of an improved, human anthrax vaccine. Eur J Epidemiol 4, 12–19 (1988). 3128450 10.1007/BF00152686

[b42] Gat O. . Characterization of Bacillus anthracis iron-regulated surface determinant (Isd) proteins containing NEAT domains. Mol Microbiol 70, 983–999 (2008). 18826411 10.1111/j.1365-2958.2008.06460.x

[b43] Gat O. . The solute-binding component of a putative Mn(II) ABC transporter (MntA) is a novel Bacillus anthracis virulence determinant. Mol Microbiol 58, 533–551 (2005). 16194238 10.1111/j.1365-2958.2005.04848.x

[b44] Janes B. K. & Stibitz S. Routine markerless gene replacement in Bacillus anthracis. Infect Immun 74, 1949–1953 (2006). 16495572 10.1128/IAI.74.3.1949-1953.2006 PMC1418658

[b45] Levy H. . Differential contribution of Bacillus anthracis toxins to pathogenicity in two animal models. Infect Immun 80, 2623–2631 (2012). 22585968 10.1128/IAI.00244-12 PMC3434592

[b46] Levy H. . The effect of deletion of the edema factor on Bacillus anthracis pathogenicity in guinea pigs and rabbits. Microb Pathog 52, 55–60 (2012). 22020310 10.1016/j.micpath.2011.10.002

[b47] Gat O., Grosfeld H. & Shafferman A. *In vitro* screen of bioinformatically selected Bacillus anthracis vaccine candidates by coupled transcription, translation, and immunoprecipitation analysis. Methods Mol Biol 375, 211–233 (2007). 17634604 10.1007/978-1-59745-388-2_11

[b48] Kobiler D. . Protective antigen as a correlative marker for anthrax in animal models. Infect Immun 74, 5871–5876 (2006). 16988266 10.1128/IAI.00792-06 PMC1594923

[b49] Weiss S., Levy H., Fisher M., Kobiler D. & Altboum Z. Involvement of TLR2 in innate response to Bacillus anthracis infection. Innate Immun 15, 43–51 (2009). 19201824 10.1177/1753425908100379

[b50] Aloni-Grinstein R., Gat O., Altboum Z., Velan B., Cohen S. & Shafferman A. Oral spore vaccine based on live attenuated nontoxinogenic Bacillus anthracis expressing recombinant mutant protective antigen. Infect Immun 73, 4043–4053 (2005). 15972492 10.1128/IAI.73.7.4043-4053.2005 PMC1168547

[b51] Shatalin K. . Bacillus anthracis-derived nitric oxide is essential for pathogen virulence and survival in macrophages. Proc Natl Acad Sci USA 105, 1009–1013 (2008). 18215992 10.1073/pnas.0710950105 PMC2242674

[b52] Cybulski R. J. Jr., Sanz P., Alem F., Stibitz S., Bull R. L. & O’Brien A. D. Four superoxide dismutases contribute to Bacillus anthracis virulence and provide spores with redundant protection from oxidative stress. Infect Immun 77, 274–285 (2009). 18955476 10.1128/IAI.00515-08 PMC2612278

[b53] McGillivray S. M. . ClpX contributes to innate defense peptide resistance and virulence phenotypes of Bacillus anthracis. J Innate Immun 1, 494–506 (2009). 20375606 10.1159/000225955 PMC2920483

[b54] Jenkins A., Cote C., Twenhafel N., Merkel T., Bozue J. & Welkos S. Role of purine biosynthesis in Bacillus anthracis pathogenesis and virulence. Infect Immun 79, 153–166 (2011). 21041498 10.1128/IAI.00925-10 PMC3019915

[b55] Kern J. & Schneewind O. BslA, the S-layer adhesin of B. anthracis, is a virulence factor for anthrax pathogenesis. Mol Microbiol 75, 324–332 (2010). 19906175 10.1111/j.1365-2958.2009.06958.x PMC2828814

[b56] Chateau A., van Schaik W., Six A., Aucher W. & Fouet A. CodY regulation is required for full virulence and heme iron acquisition in Bacillus anthracis. FASEB J . 25, 4445–4456 (2011). 21911592 10.1096/fj.11-188912

