# Supplementary material for: Next-Generation Bacillus anthracis Live Attenuated Spore Vaccine Based on the htrA- (High Temperature Requirement A) Sterne Strain
Source: Sci Rep. 2016 Jan 6;6:18908. doi: 10.1038/srep18908 (PMC4702213; doi:10.1038/srep18908)
Supplement: Supplementary Information [file srep18908-s1.pdf]

Next-Generation *Bacillus anthracis* Live Attenuated Spore Vaccine

Based on the *htrA*[-] (High Temperature Requirement A) Sterne Strain

Theodor Chitlaru, Maayan Israeli, Erez Bar-Haim, Uri Elia, Shahar Rotem, Sharon Ehrlich,
Ofer Cohen and Avigdor Shafferman

Supplementary Material

Figures S1-S4
Table S1
Table S2

Legends to Supplementary Figures and Table

Supplementary Figure S1

The mutated Sterne∆*htrA* and parental Sterne strains exhibit similar germination and sporulation profiles.

a: Germination assay of spores of the mutated strain (gray line) and parental strain (black line) demonstrate indistinguishable germination efficiency. The assay measures spectrophotometrically (every 30 seconds) the decrease in optical absorbance at a wavelength of 600 nm, characteristic of the depletion of spores from the culture. The figure depicts a representative experiment of at least 3 independent experiments which yielded indistinguishable results. **b**: Sporulation of the mutated (gray line) and parental (black line) strains in SSM cultures, measured by viable counting of bacteria before and after heat-shock (70°C for 20 minutes). Error bars indicate SD measured in at least three independent sporulation experiments. The analyses described employed the Sterne∆*htrA* IIBR-BA104K^{R} clone.

Supplementary Figure S2

Phenotypic characterization of the Sterne∆*htrA* strain.

a: Dilution drop assay of the parental Sterne, mutated Sterne ∆*htrA* (IIBR-BA104K^{R}) and the trans-complemented Sterne∆*htrA*/HtrA strains at 37°C and 42°C degrees. Note cessation of growth of the mutated strain at 42°C and full recovery of growth of the trans-complemented strain. **b**: Growth rates of the parental Sterne (black histograms), mutated Sterne ∆*htrA* (gray) and the trans-complemented ∆*htrA*/HtrA (white) strains at increasing concentrations of H_2O_2. Histograms represent means obtained in 3 independent experiments. Error bars represent SD.*, **: means were analyzed using t-test ($p<0.05$).

Supplementary Figure S3

Proliferation assay of the Sterne∆*htrA* strain in Macrophage cultures

J774.1 macrophage infection assays carried out with the parental Sterne (black histograms), mutated Sterne ∆*htrA* (gray) and the trans-complemented ∆*htrA*/HtrA (white) strains. **a:** level of bacteria released from macrophages measured by viable counts at the indicated time points. **b:** macrophage disruption evaluated by the release of LDH and expressed as percentage of total-lysis control groups of uninfected cells collected at the indicated times and processed as specified by the commercial LDH-L kit employed for the assay (see Materials and Methods). All analyses described above employed the Sterne∆*htrA* IIBR-BA104K^{R} strain. Identical results were obtained with the antibiotic marker-less strain Sterne∆*htrA* IIBR-BA106.
* t-test p value <0.05.

Supplementary Figure S4

<u>Functional assays of the LF and EF toxin-subunits produced by the SterneΔ*htrA* strain.</u>

A: LF functional assays of NBY-CO_2 supernatants of the Sterne (black curve) and SterneΔ*htrA* (gray curve) strain, based on LF-mediated macrophage lysis. Negative control (no lysis) was carried out with the non-toxinogenic and uncapsulated ΔVollum strain (pXO1$^-$; pXO2$^-$, black dotted curve) or with a SterneΔ*lef* strain (grey dotted curve)*;* this strain is specifically mutated in the *lef* gene (encoding for LF; this novel strain will be described else-were, manuscript in preparation). **B:** EF functional assays of NBY-CO_2 supernatants of the Sterne (WT, lane 3) and SterneΔhtrA (Δ*htrA*, lane 4) strain, based on EF-mediated ATP depletion in a luciferase reaction. The results are expressed as percentage of the maximal luminescence obtained in the luciferase reaction (lane 1). Positive control: reactions in which 5ng of pure preparations of EF were added (lane 2); negative control: luciferase reaction carried out in the presence of supernantant collected from a non-toxinogenic and uncapsulatedΔVollum strain (lane 5) and luciferase reaction carried out in the presence of supernantant collected from a SterneΔ*cya* strain (lane 6) specifically mutated in the *cya* gene (encoding for EF; this novel strain will be described else-were, manuscript in preparation). Error bars represent the SD calculated from 3 independent experiments. All analyses described employed the SterneΔ*htrA* IIBR-BA106 clone. *, **: Means were analyzed using t-test ($p < 0.05$).

Supplementary Figure S5

<u>PA production *in-vivo* and spore dissemination to spleens determined in guinea pigs infected SC with the Sterne, and SterneΔ*htrA* strains.</u>

Guinea pigs were infected SC with various doses of Sterne (left panels) or SterneΔ*htrA* (right panels) spores as indicated. **A**: PA levels in the serum of individual animals obtained from blood samples withdrawn 3 days post-inoculation. B: Spore viable counts in the spleens of animals at the indicated time points (days) post-infection. Each dot represents the bacterial load quantified for an individual animal; histograms represent the average value. In each of the 4 panels, the left histograms represent the infection in-put dose (marked "In-put"). None of the animals were bacteremic through-out the experiment (level of detection in blood samples: 5CFU/ml). The experiments described employed the SterneΔ*htrA* IIBR-BA104K^R clone.

Supplementary Table S1

<u>Longevity of immunological status during 52 weeks in guimea pigs vaccinated with the SterneΔ*htrA* strain</u>

Anti-PA, anti-bacteria and toxin-neutralizing (TNA) titres in blood samples of guinea pigs vaccinated SC by one dose of 10^9 or two doses of 10^8 (4 weeks apart) of SterneΔ*htrA* spores (20 animals/group). The titer values represent the geometrical mean (and STD) of the titers of the animals within same experimental group. Fifty-two weeks post-immunization, all animals were challenged with 100xLD_{50} of the fully virulent *B. anthracis* Vollum strain by IN infection.

Supplementary Figure S1

a

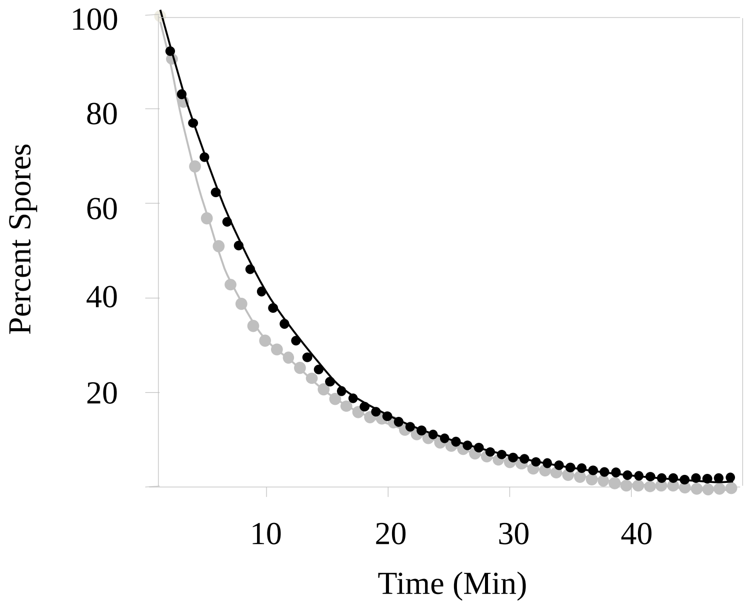

b

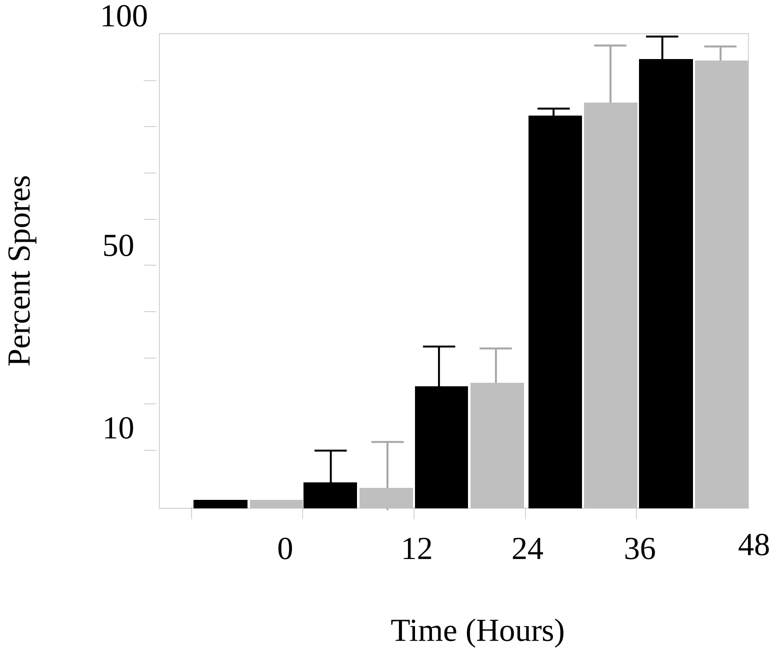

Supplementary Figure S2

a

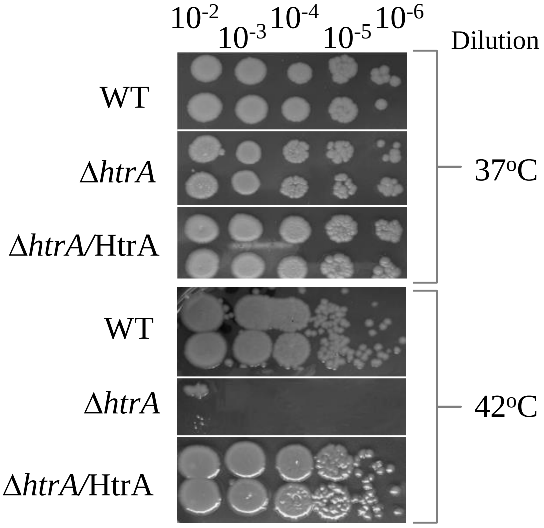

b

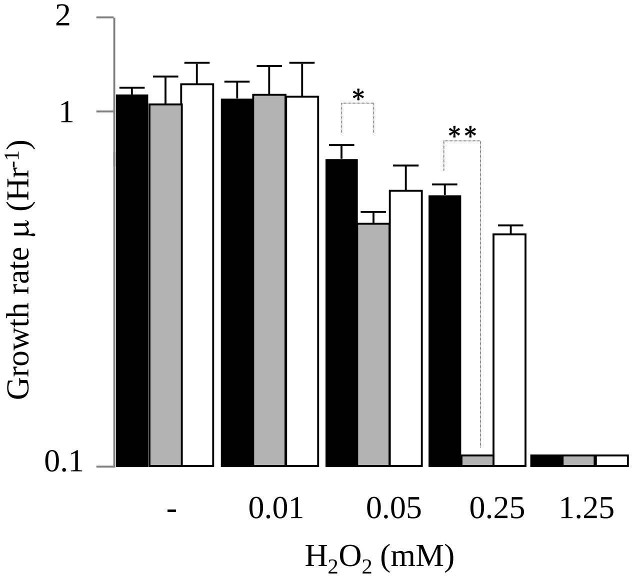

Supplementary Figure S3

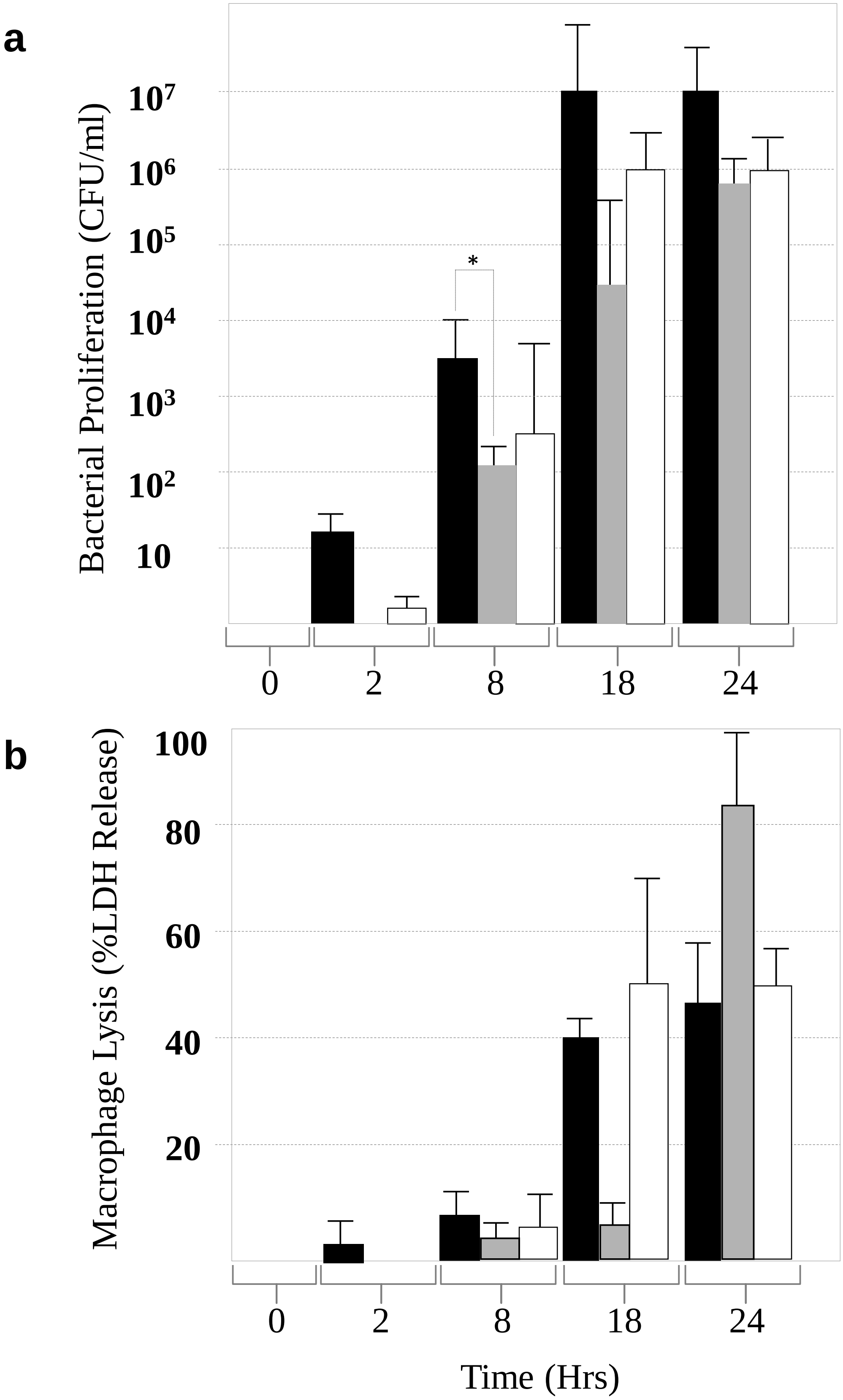

Supplementary Figure S4

a

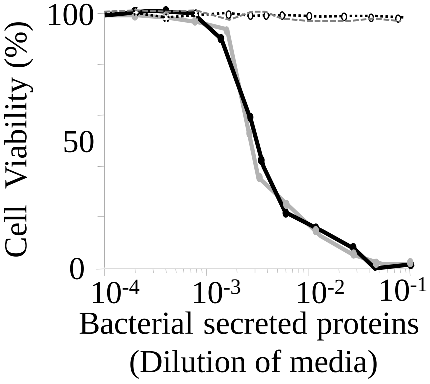

b

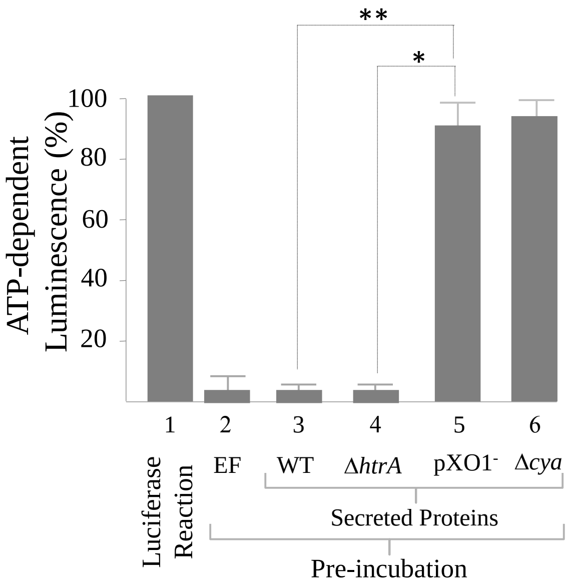

Supplementary Figure S5

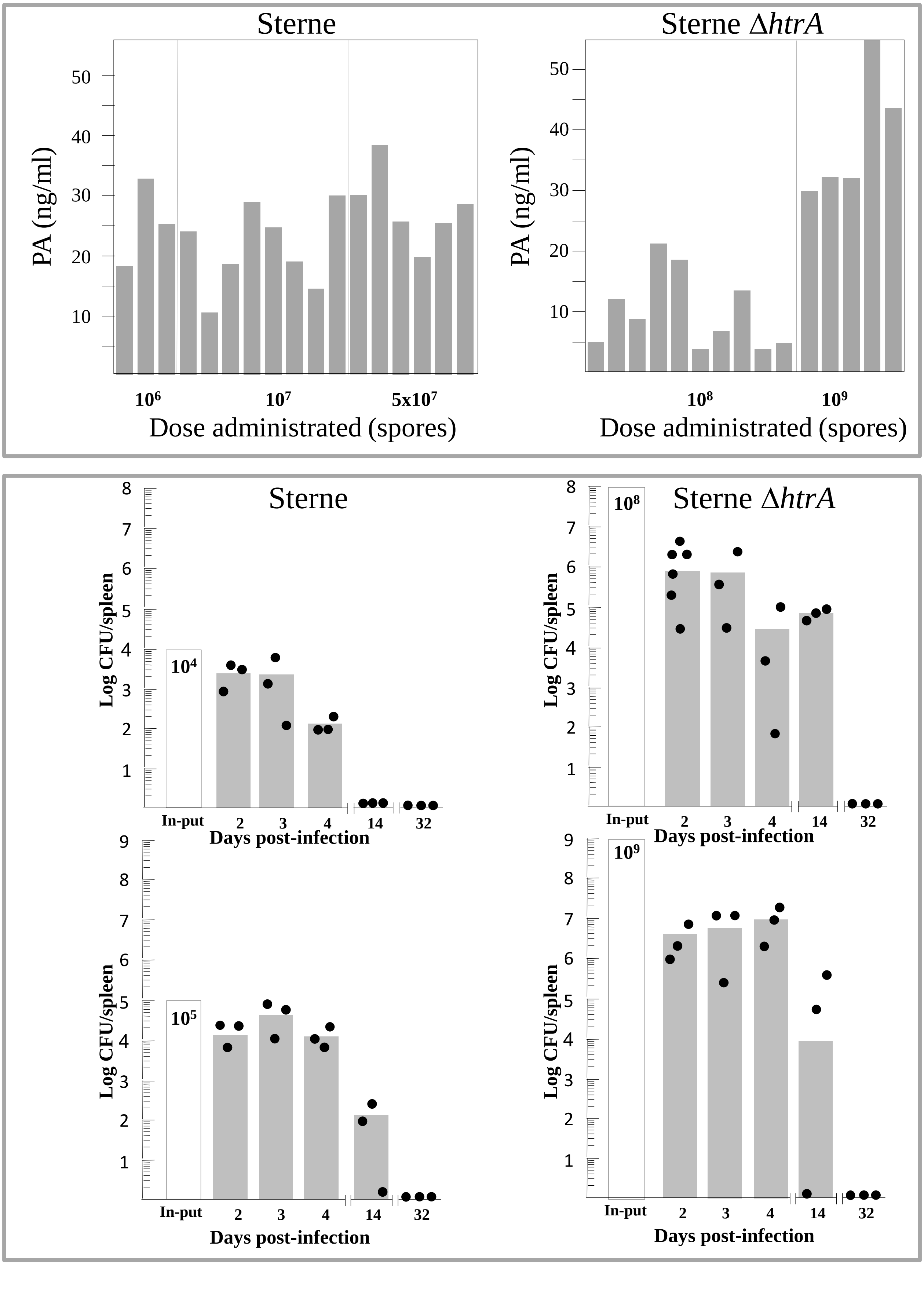

Supplementary Table S1

Longevity of immunological status during 52 weeks in guimea pigs vaccinated with the Sterne∆*htrA* strain

Time post-vaccination (weeks)	Vaccine Dose 10^9 (Single)			Vaccine Dose 10^8 (Double)		
	Anti-PA	Anti-Bacteria	TNA	Anti-PA	Anti-Bacteria	TNA
5	9.1x10^4 (5.4)	4.2x10^5 (5.6)	5.6x10^2 (2.1)	1.9 x10^5 (5.3)	8.4 x10^5 (6.1)	1.2 x10^3 (3.4)
9	1.1x10^5 (4.9)	7.6x10^5 (5.5)	9x10^2 (2.5)	2.5 x10^5 (5.5)	8.4 x10^5 (5.6)	2.0 x10^3 (3.3)
24	9 x10^4 (4.9)	2.3x10^5 (5.2)	1.6x10^3 (2.9)	8.4 x10^4 (4.6)	2.1 x10^5 (4.9)	1.1 x10^3 (3.2)
34	8.9 x10^4 (5.0)	1.2x10^5 (4.9)	1.3 x10^3 (2.9)	6.4 x10^4 (4.5)	2.3 x10^5 (5.5)	1.2 x10^3 (3.2)
50	2.1 x10^4 (4.5)	6.7 x10^4 (4.9)	7 x10^2 (2.8)	3.1 x10^4 (4.5)	8.3 x10^4 (4.9)	7.7 x10^2 (3.1)
Survival of challenge (week 52)	100% (20/20)			100% (20/20)		

Supplementary Table S2

Bacterial strains, plasmids and primers used in this study

Strains	Description/comments	Source
B. anthracis		
Vollum	Parental (wt) strain. $PA^{+}LF^{+}EF^{+}(pXO1^{+})$ $Cap^{+}(pXO2)$	ATCC14578
VollumΔ*htrA*	*htrA* nonpolar delition mutant of Vollum	Ref. 15
VollumΔ*htrA* /pHtrA	Complementation of *ΔhtrA* by *htrA* expression in trans, from pASC-HtrA	Ref. 15
Sterne	Parental (wt) strain. $PA^{+}LF^{+}EF^{+}(pXO1^{+})$ $Cap^{-}(pXO2^{-})$	IIBR stock
SterneΔ*htrA*	*htrA* nonpolar delition mutant of Sterne; Two independent clones of this strain were generated: SterneΔ*htrA*-BA104K^{R} (carrying a Kanamycin resistance gene) and SterneΔ*htrA*-BA106, devoid of antibiotic resistance. These two variants exhibit indistinguishable phenotypes.	This study
SterneΔ*htrA* /pHtrA	Complementation of *ΔhtrA* by *htrA* expression in trans, from pASC-HtrA	This study
E. coli		
DH5α	*endA1 recA1*	Clontech
GM2929	Dam13::Tn9(Cm^{R}) dcm-6	NEB

Plasmids	Source
pEO-*htrA*	Ref. 15
pASC-HtrA	Ref. 15
pEGS- *htrA*	This study
pXX-I SceI	Ref. 45

Primers	Sequence (5'→3')	Use
HTRA9	CAAGAAGGACACAAAACGTGACTATAC	Confirmation of Δ*htrA* by PCR
HTRA10C	GTATAGCCGATAAAGAAAGTCTC	
KANA1	GAAGGAATGTCTCCTGCTAAGG	
KANA2C	CTGATCGACCGGACGCAGAAG	
